# Supplementary material for: Study protocol: examining the impacts of COVID-19 mitigation measures on pregnancy and birth outcomes in Scotland—a linked administrative data study
Source: BMJ Open. 2023 Feb 15;13(2):e066293. doi: 10.1136/bmjopen-2022-066293 (PMC9933130; doi:10.1136/bmjopen-2022-066293)
Supplement: Author's manuscript [file bmjopen-2022-066293.draft_revisions.pdf]

BMJ Open is committed to open peer review. As part of this commitment we make the peer review history of every article we publish publicly available.

When an article is published we post the peer reviewers' comments and the authors' responses online. We also post the versions of the paper that were used during peer review. These are the versions that the peer review comments apply to.

The versions of the paper that follow are the versions that were submitted during the peer review process. They are not the versions of record or the final published versions. They should not be cited or distributed as the published version of this manuscript.

BMJ Open is an open access journal and the full, final, typeset and author-corrected version of record of the manuscript is available on our site with no access controls, subscription charges or pay-per-view fees (http://bmjopen.bmj.com).

If you have any questions on BMJ Open's open peer review process please email info.bmjopen@bmj.com

BMJ Open

Study Protocol: Examining the impacts of COVID-19 mitigation measures on pregnancy and birth outcomes: A linked administrative data study

Journal:	*BMJ Open*
Manuscript ID	bmjopen-2022-066293
Article Type:	Protocol
Date Submitted by the Author:	05-Jul-2022
Complete List of Authors:	Oberndorfer, Moritz; University of Glasgow MRC/CSO Social and Public Health Sciences Unit, ; Medical University of Vienna Center for Public Health<br>Henery, Paul; Public Health Scotland<br>Dundas, Ruth; University of Glasgow, MRC/CSO Social and Public Health Sciences Unit<br>Leyland, Alastair; MRC/CSO Social and Public Health Sciences Unit, University of Glasgow<br>Paranjothy, Shantini; University of Aberdeen, Health Data Science Research Centre<br>Stock, Sarah; The University of Edinburgh Usher Institute of Population Health Sciences and Informatics, Centre for Medical Informatics; Public Health Scotland,<br>Wood, Rachael; NHS National Services Scotland, Information Services Division; University of Edinburgh, Child Life and Health<br>Nelson, Scott; University of Glasgow, School of Medicine<br>Kearns, Rachel; University of Glasgow School of Medicine Dentistry and Nursing<br>Pearce, Anna; University of Glasgow MRC/CSO Social and Public Health Sciences Unit
Keywords:	EPIDEMIOLOGY, PUBLIC HEALTH, COVID-19, OBSTETRICS

SCHOLARONE™
Manuscripts

Study Protocol:

Examining the impacts of COVID-19 mitigation measures on pregnancy and birth outcomes: A linked administrative data study

Authors: Moritz Oberndorfer[1,2], Paul M. Henery[3], Ruth Dundas[2], Alastair H. Leyland[2], Shantini Paranjothy[4], Sarah J. Stock[5,6], Rachael Wood[7,8], Scott M. Nelson[9], Rachel Kearns[9], Anna Pearce[2]

[1] Department of Social and Preventive Medicine, Center for Public Health, Medical University of Vienna, Vienna, Austria.

[2] MRC/CSO Social and Public Health Sciences Unit, University of Glasgow, Glasgow, UK

[3] Public Health Scotland, Edinburgh, UK

[4] Aberdeen Centre for Health Data Science, University of Aberdeen, UK

[5] The University of Edinburgh Usher Institute of Population Health Sciences and Informatics, Centre for Medical Informatics, Edinburgh, UK

[6] Public Health Scotland, Edinburgh, UK

[7] NHS National Services Scotland, Information Services Division, Edinburgh, UK

[8] Child Life and Health, University of Edinburgh, Edinburgh, UK

[9] School of Medicine Dentistry and Nursing, University of Glasgow, Glasgow, UK

Corresponding author:

Moritz Oberndorfer

Department of Social and Preventive Medicine, Center for Public Health, Medical University of Vienna

Kinderspitalgasse 15/1, 1090, Vienna, Austria

E-mail address: moritz.oberndorfer@muv.ac.at

Tel.: 0043 1 40160 34891

MRC/CSO Social and Public Health Sciences Unit, University of Glasgow

Berkeley Square, 99 Berkeley Street, Glasgow, G3 7HR, UK

E-mail address: moritz.oberndorfer@glasgow.ac.uk

Word count: ~3700

For peer review only - http://bmjopen.bmj.com/site/about/guidelines.xhtml

Abstract

Introduction

This protocol outlines plans to test the wider impacts of the COVID-19 pandemic on pregnancy and birth outcomes and inequalities in Scotland.

Method & analysis

We will analyse Scottish linked administrative data for pregnancies and births before (March 2010-March 2020) and during (April 2020-October 2020) the pandemic. The Community Health Index database will be used to link the National Records of Scotland Births and the Scottish Morbidity Record 02. The data will include about 500,000 mother-child pairs. We will investigate population-level changes in maternal behaviour (smoking at ante-natal care booking, infant feeding on discharge), pregnancy and birth outcomes (birth weight, preterm birth, Apgar score, stillbirth, neonatal death, pre-eclampsia), and service use (mode of delivery, mode of anaesthesia, neonatal unit admission) during the COVID-19 pandemic using two analytical approaches. First, we will estimate interrupted times series regression models to describe changes in outcomes comparing pre-pandemic with pandemic periods. Second, we will analyse the effect of COVID-19 mitigation measures on our outcomes in more detail by creating cumulative exposure variables for each mother-child pair using the Oxford Covid-19 Government Response Tracker. Thus, estimating a potential dose-response relationship between exposure to mitigation measures and our outcomes of interest as well as potential effect moderation by timing of exposure during pregnancy. Finally, we will assess inequalities in the effect of cumulative exposure to lockdown measures on outcomes using several axes of inequality: ethnicity/mother's country of birth, area deprivation (Scottish Index of Multiple Deprivation), urban-rural classification of residence, number of previous children, maternal social position (NS-SEC), and parental relationship status.

Ethics and dissemination

NHS Scotland Public Benefit and Privacy Panel for Health and Social Care (HSC-PBPP) scrutinised and approved the use of these data (1920-0097). Results of this study will be disseminated to the research community, practitioners, policy makers, and the wider public.

For peer review only - http://bmjopen.bmj.com/site/about/guidelines.xhtml

Strengths and limitations of the study

- We will use population-wide administrative data covering all mother-child pairs for children born in Scotland between March 2010 and October 2020 to study how population-level pregnancy and birth outcomes changed during the COVID-19 pandemic.
- Using the Stringency Index recorded by the Oxford Covid-19 Government Response Tracker (OxCGRT), we are able to calculate an individual level of cumulative exposure to pandemic mitigation measures for each mother-child pair in our data.
- Our effect estimates will be biased if unmeasured factors changed routine data collection (patterns of missing or misclassified data), or – for post-natal outcomes – if the characteristics of livebirths during the COVID-19 pandemic had changed in a way that is associated with our outcomes of interest.

For peer review only

For peer review only - http://bmjopen.bmj.com/site/about/guidelines.xhtml

Introduction

Early on in the Coronavirus disease 2019 (COVID-19) pandemic, concerns were raised about the widespread and unequal impacts of social mitigation measures on health and the social determinants of health[1] including for children and families[2,3]. In this protocol, we focus on parents and children during pregnancy and at birth. Figure 1 outlines three key, interlinked mechanisms through which the wider pandemic (distinct from the risks of contracting the virus) may have had negative (and sometime positive) effects on this group. The first surrounds changes to health services. Pregnant women were identified as being particularly vulnerable to the severe effects of COVID-19, prompting early advice from the NHS to adopt social distancing. This, alongside the strain put on health services by the wider pandemic, meant that the services and support for pregnant and new mothers dramatically changed.[4] Non-urgent procedures and contacts were cancelled, and resources diverted from elective to critical care. Guidance and services were quickly innovated to support new families, including the use of virtual technologies to provide health appointments, antenatal classes and hospital tours; mothers were supported to self-monitor glucose, urine and blood pressure at home; the provision of clinics in community settings increased. Partners were allowed in hospital only for the last stages of labour and no other visitors were permitted at any point during the hospital stay.[4] Although many of these restrictions have since eased, the services that young families receive have not fully returned to normal and uncertainty remains.

The second mechanism refers to psychosocial factors. Negative impacts of lockdown on mental wellbeing have been documented, alongside increases in job loss, job insecurity and universal credit claims among the adult population.[5–9] Profound changes to services and birthing plans, the disruption of feeding intentions and expectations around parenthood, and anxiety around catching the virus, have led to increased uncertainty and feelings of isolation among pregnant mothers and new families, causing psychological distress.[10,11]

Third, in the general population many health behaviours were affected, with diets becoming less healthy both in terms of quality and quantity[12] and alcohol consumption increasing, particularly among groups who were already high consumers[13]. Conversely, smoking has declined[5] and it has been hypothesised that working from home, lower exposure to air pollutants, and better hygiene habits may have benefited foetal development and health[14]. Hospital support for breastfeeding immediately after birth has remained[10], and breastfeeding rates upon discharge have not necessarily been affected[15]. However, lack of support from friends and family, mother and baby groups, and health professionals has been highlighted as a barrier to feeding after returning home.[10]

For peer review only - http://bmjopen.bmj.com/site/about/guidelines.xhtml

Our aim is not to test these different mechanisms, but to first establish the overall impacts of the pandemic on various mother and infant outcomes, and inequalities in these outcomes, in Scotland. This will provide a better understanding of potential future health challenges and to inform responses to the ongoing and any future pandemics. A comprehensive investigation of pregnancy and birth outcomes, in Scotland during March-May 2020 (compared to two years previous), found that some procedural outcomes showed changes in the expected direction (e.g. length of hospital stay decreased), but few changes in maternal and infant health outcomes.[15] Few signs of negative impacts (in high income countries) have also been detected in international systematic reviews and meta-analyses[14,16], with the exception of maternal mental health[16]. However, while the overall picture is positive, it remains plausible that these studies have overlooked differential effects occurring at the sub-group level. In the case of the three of proposed mechanisms discussed above, it is likely that some groups, including those from less advantaged social circumstances, first time mothers, and ethnic minority, groups have fared worse than others.[1,17] There are also some indications in the limited evidence base that birth and pregnancy have worsened from some groups and not others. For example, there was no change in stillbirths in England overall, but rates had increased in North England.[18] In Canada, new-born readmission rates among first time mothers were higher after the pandemic, while multiparous women were less likely to experience pre-term birth rates, low Apgar scores and hospital readmissions.[19] Furthermore, it is possible that early studies considering outcomes only at the very start of the pandemic may have overlooked impacts on expectant mothers who were exposed to social mitigation measures for longer durations of pregnancy.

This protocol outlines plans to estimate the wider impacts of the COVID-19 pandemic on pregnancy and birth outcomes and inequalities in Scotland. We will take a natural experiment approach to identify any step change trends in outcomes at the start of the pandemic, limiting our analyses to pregnancies which were conceived before the pandemic, to avoid introducing bias due to the changing socio-demographic characteristics of conceptions which occurred after the start of the pandemic[20]. Furthermore, we will use the stringency index (which measures the strictness of policies that primarily restrict people's behaviour) to consider the cumulative effects of social mitigation measures across pregnancy, by comparing cohorts with different lengths or intensity of exposure. Additionally, we will consider timing of exposure, as it is possible that, for some outcomes, any impacts of the stresses related to the pandemic and social mitigation measures might be greater during some trimesters of pregnancy than others[21]. Finally, we will investigate whether exposure to mitigation measures had a differential effect on our outcomes across several axes of inequalities.

For peer review only - http://bmjopen.bmj.com/site/about/guidelines.xhtml

Methods

Patient and public involvement

This secondary analysis of data will not directly involve the public or patients. Findings will be disseminated to relevant health professionals and interest groups to maximise benefits for service provision throughout Scotland.

Study design and population

We will employ two analytical approaches, each informed by the logic model in Figure 1. In our first analytical approach, we will provide, using interrupted time series regression models, a descriptive visualisation of how outcome variables changed between pre-pandemic (March 2010-March 2020) and pandemic (April 2020-October 2020). Births from November 2020 onwards will be excluded from our regression analysis since the majority were conceived during lockdown, and the pandemic and its socioeconomic consequences might have affected fertility and thereby the characteristics of new families in ways that we cannot fully account for.[20,22] In this first approach we will ignore variation in exposure to mitigation measures during pregnancy and at birth as we aim to estimate the average population level impact of COVID-19 mitigation measures on pregnancy and birth outcomes.

In our second analytical approach, we will investigate the relationship between the outcomes and exposure to mitigation measures in more detail. As the intensity, duration, and timing of exposure to COVID-19 mitigation measures is dependent on the date of conception and duration of pregnancy, each mother and child pair will be given an individually calculated level of cumulative exposure to mitigation measures in Scotland using the Stringency Index created by the Oxford Covid-19 Government Response Tracker (OxCGRT)[23]. This allows us to estimate a potential dose-response relationship between exposure to mitigation measures as well as potential effect moderation by timing of exposure (focussing on trimesters).

Databases

We will use linked data from the below datasets:

National Records of Scotland (NRS) Births: The NRS holds information on all births registered in Scotland since 1975. These records include information on date and location of the birth and details of the registered parent(s), including their marital/relationship status and their occupational status.

Scottish Morbidity Record 02 (SMR02): SMR02 records all maternity and infant inpatient and day case episodes in Scotland. Around 50% episodes relate to births and it was these records that were

For peer review only - http://bmjopen.bmj.com/site/about/guidelines.xhtml

requested for the purposes of the cohort. These include demographic characteristics and information relating to the birth and clinical management.

National Records Scotland (NRS) and The Scottish Stillbirths and Infant Deaths Survey (SSBIDS): register of all births, stillbirths, and infant (including neonatal) deaths.

Scottish Birth Records (SBR): all records of a baby's neonatal care in Scotland

Community Health Index (CHI) Database: This contains a unique identifier for all NHS users in Scotland (~99% of population) and is used to link the above datasets.

Outcomes

We chose outcomes that could feasibly be affected by social mitigation measures (Figure 1 logic model) and for their relevance for subsequent child and adult health. We grouped them into maternal behaviours, birth and pregnancy outcomes, and service use.

Maternal behaviours: Smoking in pregnancy, usually measured during the ante-natal care booking (~8 -12 weeks of pregnancy) supplemented by information collected at any subsequent ante-natal appointments (yes; no). Infant feeding at discharge from hospital (breastfeeding - yes; no).

Birth and pregnancy outcomes: Birthweight in grams (continuous variable); low birthweight <2500g and high birthweight >4000g. Similarly, gestational age will be considered as a continuous variable and categorised to identify preterm birth (delivery before 37^{+0} weeks of gestation) and late gestational age (≥42^{+0} weeks). We will carry out sensitivity analyses differentiating different degrees of prematurity (extremely preterm: <28^{+0} weeks; very preterm: 28^{+0} to 31^{+6} weeks; moderate to late preterm: 32^{+0} to 36^{+6} weeks) and low birthweight (extremely low: <1000g; very low: 1000 to 1499g; low: 1500g to 2499g), since previous research has found delays in extreme prematurity which only manifest in reductions in 'very premature'[24]. Additionally, we will analyse birthweight standardised for gestational age and consequently small for gestational age (SGA) as well as large for gestational age (LGA) as outcomes to explicitly focus on fetal growth. The Apgar score, measured within the first five minutes after delivery, assesses five characteristics (heart rate, respiratory effort, muscle tone, reflex irritability, colour), and can be dichotomised to measure good to excellent infant health (score of 7 or higher[25]). Finally, we will examine preeclampsia.

Health services use: Mode of delivery will consist of four categories (spontaneous vaginal, assisted vaginal, planned caesarean, emergency caesarean), mode of anaesthesia (spinal, general anaesthesia, epidural), and neonatal unit admissions.

For peer review only - http://bmjopen.bmj.com/site/about/guidelines.xhtml

Most of the outcomes under examination are relatively common (e.g., rate of preterm births is 65 per 1,000). The least common are stillbirths (5 per 1,000) and low birth weight (20 per 1,000). With 27,100 births occurred during the pandemic period (April 2020-October 2020[26]), these outcomes are relatively infrequent.

Secondary outcomes: We will also consider changes before/during the pandemic in the following secondary outcomes: miscarriage (loss of baby during first 23 weeks of pregnancy), stillbirths (loss of a baby after 24 weeks of gestation) and neonatal deaths (first 28 days after delivery). Some of these outcomes are very rare (e.g. neonatal deaths is <0.2%) and so may only be used to identify bias, with outcome data not reported. Analysis of changes in our secondary outcomes will inform our analysis of post-natal outcomes. If, for example, rates of stillbirths and miscarriages were higher during the pandemic compared to pre-pandemic periods, we expect the pandemic to have an indirect positive effect on post-natal outcomes via this selection mechanism.

Exposure

For our first approach – the interrupted time series analysis - we will use dummy variables to indicate whether the outcome (measured at booking or at birth, depending on the outcome) was observed during pre-pandemic (before first lockdown measures in March 2020) or pandemic periods (April 2020 to October 2020). In light of substantial compositional change in maternal characteristics observed in Scotland and their potential effects on our outcomes[20,27], we will additionally include November and December 2020 in our visualisations (if this data becomes available at time of analysis), but restrict our modelling to observations up until October 2020.

For our second analysis, we will calculate an individual level of cumulative exposure for each mother-child pair using the OxCGRT. The OxCGRT has recorded government responses to the COVID-19 pandemic. Methodological details of the OxCGRT have been described elsewhere.[23] As a measure of the stringency of lockdown measures, we will use the OxCGRT Stringency Index (SI) which comprises 9 different indicators (school closing, workplace closing, cancel public events, restriction on gathering size, close public transport, stay at-home order requirements, restrictions on internal movement, restriction on international travel, public health campaigns). The SI ranges from 0 to 100 and has been recorded daily since January 2020. Cumulative exposure to lockdown measures will be calculated by the sum of weekly averages of SI during pregnancy and up until the occurrence of the outcome. Figure 2 visualises the level of cumulative exposure for mother-child pairs by week of conception for different gestational ages. As raised in the introduction, it is possible that timing of exposure to social mitigation matters. We will therefore also examine cumulative exposure within each trimester of pregnancy.

For peer review only - http://bmjopen.bmj.com/site/about/guidelines.xhtml

Population characteristics and confounding factors

All models will include dummy variables indicating which month the outcomes were observed (with January being the reference) to account for seasonality and the correlation between month of birth and cumulative exposure.

In the second analytic approach, duration of pregnancy will be associated with cumulative exposure to mitigations measures and the outcomes and will thus be adjusted for. Yet, duration of pregnancy may not only be a confounder of the exposure-outcome relationship for post-natal outcomes, but also a mediator as exposure to the pandemic might affect gestational age (e.g., by changing maternal behaviour or health services). Mediator analyses are not part of our study, but as gestational age is one of our outcomes under investigation, model interpretation for post-natal outcomes will be informed by the estimated effect size of our cumulative exposure on gestational age.

We will also adjust for variables that are associated with the outcome but not with the exposure – to take account of potential time trends in outcomes, including, where sufficiently complete: maternal age, maternal occupational class measured by NS-SEC, ethnicity of mother, sex of the baby, Scottish Index of Multiple Deprivation, and urban-rural classification of residence. Informed by previous work[28], we expect a large proportion of missing information on maternal ethnicity (around 50%) but high completeness (>90%) in the other variables.

For post-natal outcomes (birthweight, Apgar score, neonatal death, infant feeding on discharge, mode of delivery, mode of anaesthesia, neonatal unit admission), we consider change in prevalence of miscarriage, pregnancy terminations, stillbirths, and maternal emigration behaviour during pregnancy due to our exposure variable as potential mediators of the exposure-outcome relationship rather than confounders. However, as the pandemic might increase likelihood of these events as a function of vulnerability to our exposure, this pathway could potentially result in a positive effect of the exposure on outcomes (for example birthweight). Blocking this pathway from exposure to outcome in our analysis will avoid potentially counteracting causes that might deceptively lead to attenuated effects. This will be partially achieved by the control variables introduced in Model (3), as we expect these characteristics of mother-child pairs to be associated with a potential change in likelihood of these events due to the pandemic.

Impacts on inequalities

In both approaches, several axes of inequality will be examined to consider whether the impacts of the pandemic have been differential: ethnicity/mother's country of birth (depending on completeness and available sample size), area deprivation (Scottish Index of Multiple Deprivation (SIMD)), urban-rurality classification of residence, first time mothers, maternal social position (National Statistics

For peer review only - http://bmjopen.bmj.com/site/about/guidelines.xhtml

Socioeconomic Classification (NS-SEC)), and relationship status of parents (sole registrations, separated, cohabitating, married). We will measure both absolute and relative inequalities.

Statistical analysis

In the first approach, we will use interrupted time series regression models to describe time trends in the outcomes. Therefore, we will constrain this analysis to linear functions of time. Covariates in these models will be time (weeks, or months) since first date of collected data, a dummy variable indicating whether an observation belongs to the exposed or unexposed group, an interaction between time and the exposure dummy variable, and dummy variables indicating in which month the outcome was observed with January being the reference month. Our data is structured by two levels: mother-child pairs nested within small geographic areas. Therefore, we will use multilevel modelling throughout our regression analysis. Model (1) exemplarily shows the formal specification for the continuous outcome birth weight y_{ij} of mother-child pair *i* nested within small area (data zone) *j*.

$$y_{ij} = \beta_0 + \beta_1 week_{ij} + \beta_2 exposed_{ij} + \beta_3 week_{ij} X exposed_{ij} + \sum_{t=1}^{11} \beta_t month_{ij} + u_{0j} + \varepsilon_{0ij}, \quad u_{0j} \sim N\left(0, \sigma_{u0}^2\right), \ \varepsilon_{0ij} \sim N\left(0, \sigma_{e0}^2\right) \quad (1)$$

In the second approach, the exposure is the cumulative Stringency Index and we will adjust for potential confounders. As an example, we formally describe our models for the continuous outcome birthweight below.

$$y_{ij} = \beta_0 + \beta_1 SI_{ij} + \beta_2 DoP_{ij} + \sum_{t=1}^{11} \beta_t month_{ij} + u_{0j} + \varepsilon_{0ij}, \quad u_{0j} \sim N\left(0, \sigma_{u0}^2\right), \ \varepsilon_{0ij} \sim N\left(0, \sigma_{e0}^2\right) \quad (2)$$

Model (2) presents our most parsimonious model specification, wherein y_{ij} is birthweight (in grams) measured for mother-child pair *i* in data zone *j*, SI_{ij} is the sum of weekly average Stringency Index during pregnancy of mother-child pair *ij,* DoP_{ij} is the duration of pregnancy (in weeks) for mother-child pair *ij,* and $month_{ij}$ is a dummy variable that indicates in which month birth was given with January being the reference category. In Model (3), we further include the neutral control variables maternal age, sex of baby, maternal NS-SEC, SIMD, and urban-rural classification of residence. In case there is considerable missing information in a neutral control variable, we will omit it from our models as the risk of bias induced by missing not at random likely outweighs the potential gains of a neutral control.

$$y_{ij} = \beta_0 + \beta_1 SI_{ij} + \beta_2 DoP_{ij} + \sum_{t=1}^{11} \beta_t month_{ij} + \beta_3 age_{ij} + \beta_4 sex_{ij} + \beta_5 NSSEC_{ij} + \beta_6 SIMD_{ij} + \beta_7 urban_{ij} + u_{0j} + \varepsilon_{0ij}, \quad u_{0j} \sim N\left(0, \sigma_{u0}^2\right), \ \varepsilon_{0ij} \sim N\left(0, \sigma_{e0}^2\right) \quad (3)$$

Moreover, for post-natal outcomes, we will explore whether timing of exposure matters by including variables for cumulative exposure during each trimester of pregnancy as shown in Model (4). Wherein

For peer review only - http://bmjopen.bmj.com/site/about/guidelines.xhtml

$SI1_{ij}$, $SI2_{ij}$, $SI3_{ij}$ is the sum of weekly average Stringency Index during the first, second, and third trimester of pregnancy of mother-child pair *ij.* Figure 3 illustrates cumulative exposure to mitigation measures during each trimester. As our data do not include cohorts that experienced high levels of exposure during their first trimester and low exposure during their third trimester, we will only test differences in the effect of exposure during the third and second trimester[21].

$$y_{ij} = \beta_0 + \beta_1 SI1_{ij} + \beta_2 SI2_{ij} + \beta_3 SI3_{ij} + \beta_4 DoP_{ij} + \sum_{t=1}^{11} \beta_t month_{ij} + \beta_5 age_{ij} + \beta_6 sex_{ij} + \beta_7 NSSEC_{ij} + \beta_8 SIMD_{ij} + \beta_9 urban_{ij} + u_{0j} + \varepsilon_{0ij}, \;\; u_{0j} \sim N\left(0,\sigma_{u0}^2\right), \;\; \varepsilon_{0ij} \sim N\left(0,\sigma_{e0}^2\right) \quad (4)$$

To help with the interpretation of our results, we will present estimated values for hypothetical plausible levels of cumulative exposure (i.e. exposure to 3,6,9 months of a Stringency Index of 50,60,70, etc.). In addition, we will present the estimated average outcome values at specific time points. Note that no mother-child pair in our data has experienced a different level of exposure at the same date of birth and length of gestation.

We will examine differential effects by including interaction terms with the modifying variables (see inequalities section). Where interactions appear to be meaningful, we will stratify the models. Inequalities in effect sizes will be examined by comparing average effects between levels of moderating variables. In Model (5), we exemplarily show the specification of such a model for inequalities in the effect of our exposure variable along parental NS-SEC.

$$y_{ij} = \beta_0 + \beta_1 SI_{ij} + \beta_2 DoP_{ij} + \sum_{t=1}^{11} \beta_t month_{ij} + \beta_3 age_{ij} + \beta_4 sex_{ij} + \beta_5 NSSEC_{ij} + \beta_6 SIMD_{ij} + \beta_7 urban_{ij} + \beta_8 \, SI_{ij} \, x \, NSSEC_{ij} + u_{0j} + \varepsilon_{0ij}, \;\; u_{0j} \sim N\left(0,\sigma_{u0}^2\right), \;\; \varepsilon_{0ij} \sim N\left(0,\sigma_{e0}^2\right) \quad (5)$$

Multilevel linear models will be used for continuous outcomes (as appropriate for the distribution of outcome data), with multilevel binary and multinomial logistic regression models used for binary and categorical outcomes respectively. All models will be estimated by maximum likelihood. We will derive prevalence ratios and absolute differences from model estimates.

For peer review only - http://bmjopen.bmj.com/site/about/guidelines.xhtml

Sensitivity analysis

We will explore non-linearity in the effect of cumulative exposure to lockdown measures by re-estimating our models with a quadratic functional form of the exposure variable as well as a semi-parametric specification, in which we use quintiles of the exposure variable as cut-offs to form discrete levels of cumulative exposure. We will repeat analyses limited to singleton births. Additionally, we will analyse induced and spontaneous preterm births separately (if sample size is sufficient). Depending on the partnership status of parents at birth registration, we will also have information on paternal NS-SEC. We will conduct sensitivity analyses in which we exchange maternal with paternal NS-SEC where available, as well as taking the higher occupational class in the household.

As noted previously, excluding births conceived during lockdowns will reduce unmeasured or residual confounding due to changed sociodemographic parental characteristics likely associated with the outcomes.[20] However, changes in the likelihood of miscarriage, pregnancy terminations, stillbirths, neonatal deaths and maternal emigration behaviour during pregnancy may still introduce bias for post-natal outcomes. We will explore this by analysing time trends for available variables (stillbirth, miscarriage, neonatal death) using interrupted time series regression as described above. If this analysis suggests that our exposure-outcome relationship is susceptible to such potential selection bias, we will further control (where possible) for variables that are likely associated with miscarriage, pregnancy terminations, stillbirths, and maternal emigration behaviour during pregnancy as well as the outcomes (but not affected by the exposure).

Finally, we will explore unmeasured confounding by splitting our data in multiple unexposed comparison groups (April to October for each year between 2010 and 2019).[29] Systematic differences in our outcomes between unexposed groups conditional on the covariates listed above will be tested by estimating the effect of dummy variables indicative of which comparison group a mother-child pair belongs to using regression analyses. Systematic differences in the outcomes between unexposed comparison groups even after adjusting for our set of covariates will reveal whether there is potential unmeasured confounding in respect to the effect of our cumulative exposure variable on the outcomes. The exposure-outcome relationship will then be estimated using varying sets of unexposed comparison groups against the exposed group of mother-child pairs (April to October 2020). Resulting effect sizes will be shown in forest plots and a pooled effect will be estimated by random-effects meta-analysis. In case unexposed comparison groups indeed differ in respect to our outcomes after covariate adjustment, results of this pooled analysis will be interpreted in light of unexplainable differences between unexposed groups.

For peer review only

For peer review only - http://bmjopen.bmj.com/site/about/guidelines.xhtml

Sample size

Our sample consists of all child and mother pairs for children born in Scotland between March 2010 and October 2020. Sample size is expected to be *n*~500,000 mother-child pairs (estimated based on an average of 50,000 births per annum).

Missing data

We will document levels of missing data in all variables of interest, over time and according to the potential effect moderators, for two reasons. First, understanding how data collection was impacted during the early stages of the pandemic can inform responses to future pandemics. Second, changes in patterns of missingness in the data, due to the pandemic, could introduce bias. In case of considerable levels of missing data, item missingness will be addressed using multiple imputation by chained equations.

Ethics and dissemination

Use of the data have been approved by the Public Benefit and Privacy Panel for Health and Social Care. Results of this research will be disseminated in peer reviewed presentations at public health national and international conferences and open access, peer reviewed journal articles. We will produce a briefing paper for policy-makers and practitioners and will work with in-house press advisors to ensure visibility in newspapers, radio etc. and on our COVID-19 Unit webpage.

Authors' contributions

RD, AHL, MO and AP conceived the study. SMN obtained the data and associated approvals. All authors contributed to the conception of the study design. MO, AP and PMH drafted the study protocol. All authors provided critical feedback on the draft manuscript and approved the final version.

Funding statement

The work of the authors was supported by the Medical Research Council (MC_UU_00022/2) and the Scottish Government Chief Scientist Office (SPHSU17). AP also receives support from the Wellcome Trust (205412/Z/16/Z). MO is also supported by the Marietta-Blau Scholarship (MPC-2021-00178; funded by the Austrian Federal Ministry of Education, Science and Research). AHL, AP, and RD are members of, and receive support from, the UK Prevention Research Partnership Maternal and Child Health Network (MR/S037608/1).

Competing interests statement

The authors declare that they have no competing interest.

For peer review only - http://bmjopen.bmj.com/site/about/guidelines.xhtml

For peer review only

Word Count

~3700

Figure captions

Figure 1: *Logic model demonstrating the mechanisms and moderators of the wider impacts of the pandemic on pregnancy and birth outcomes*

Figure 2: *Visual description of our exposure variable. Sum of weekly average Stringency Index (left vertical axis) during pregnancy for each week of conception (42 weeks of gestation being the top line and 32 weeks of gestation being the bottom line) between January 2018 and December 2020. Level of cumulative exposure is shown for gestational age (32 to 42 weeks). Crude weekly average Stringency Index for Scotland is shown in brown (right vertical axis). Conceptions after March 2020 (indicated by the dashed red line) are excluded from our analyses.*

Figure 3: *Exposed groups under investigation. Cumulative level of exposures presented here are the sum of weekly averages of the Stringency Index within each trimester up to month of birth. In this figure, conceptions and births are assumed to occur on the first of each month with equal gestational age. Note that, in the analyses, cumulative exposure is calculated for each mother-child pair individually and, thus, these exposure levels do not match those in Figure 2.*

References

1. Bambra C, Riordan R, Ford J, Matthews F. The COVID-19 pandemic and health inequalities. *J Epidemiol Community Health*. Published online June 12, 2020. doi:10.1136/jech-2020-214401

2. Hefferon C, Taylor C, Bennett D, et al. Priorities for the child public health response to the COVID-19 pandemic recovery in England. *Archives of Disease in Childhood*. 2021;106(6):533-538. doi:10.1136/archdischild-2020-320214

3. Goldfeld S, O'Connor E, Sung V, et al. Potential indirect impacts of the COVID-19 pandemic on children: a narrative review using a community child health lens. *Med J Aust*. 2022;216(7). doi:10.5694/mja2.51368

4. Jardine J, Relph S, Magee L, et al. Maternity services in the UK during the coronavirus disease 2019 pandemic: a national survey of modifications to standard care. *BJOG: An International Journal of Obstetrics & Gynaecology*. 2021;128(5):880-889. doi:10.1111/1471-0528.16547

5. Niedzwiedz CL, Green MJ, Benzeval M, et al. Mental health and health behaviours before and during the initial phase of the COVID-19 lockdown: longitudinal analyses of the UK Household Longitudinal Study. *J Epidemiol Community Health*. 2021;75(3):224-231. doi:10.1136/jech-2020-215060

6. Oberndorfer M, Dorner TE, Brunnmayr M, Berger K, Dugandzic B, Bach M. Health-related and socio-economic burden of the COVID-19 pandemic in Vienna. *Health & Social Care in the Community*. 2021;n/a(n/a). doi:10.1111/hsc.13485

7. Serrano-Alarcón M, Kentikelenis A, Mckee M, Stuckler D. Impact of COVID-19 lockdowns on mental health: Evidence from a quasi-natural experiment in England and Scotland. *Health Econ*. Published online November 12, 2021:10.1002/hec.4453. doi:10.1002/hec.4453

8. Chandola T, Kumari M, Booker CL, Benzeval M. The mental health impact of COVID-19 and lockdown-related stressors among adults in the UK. *Psychol Med*. Published online December 7, 2020:1-10. doi:10.1017/S0033291720005048

For peer review only - http://bmjopen.bmj.com/site/about/guidelines.xhtml

9. Patel K, Robertson E, Kwong ASF, et al. Psychological Distress Before and During the COVID-19 Pandemic Among Adults in the United Kingdom Based on Coordinated Analyses of 11 Longitudinal Studies. *JAMA Network Open*. 2022;5(4):e227629. doi:10.1001/jamanetworkopen.2022.7629

10. Vazquez-Vazquez A, Dib S, Rougeaux E, Wells JC, Fewtrell MS. The impact of the Covid-19 lockdown on the experiences and feeding practices of new mothers in the UK: Preliminary data from the COVID-19 New Mum Study. *Appetite*. 2021;156:104985. doi:10.1016/j.appet.2020.104985

11. Fallon V, Davies SM, Silverio SA, Jackson L, De Pascalis L, Harrold JA. Psychosocial experiences of postnatal women during the COVID-19 pandemic. A UK-wide study of prevalence rates and risk factors for clinically relevant depression and anxiety. *J Psychiatr Res*. 2021;136:157-166. doi:10.1016/j.jpsychires.2021.01.048

12. McBride E, Arden MA, Chater A, Chilcot J. The impact of COVID-19 on health behaviour, well-being, and long-term physical health. *Br J Health Psychol*. 2021;26(2):259-270. doi:10.1111/bjhp.12520

13. Public Health England. Monitoring alcohol consumption and harm during the COVID-19 pandemic. Published online 2021.

14. Yang J, D'Souza R, Kharrat A, et al. COVID-19 pandemic and population-level pregnancy and neonatal outcomes: a living systematic review and meta-analysis. *Acta Obstet Gynecol Scand*. 2021;100(10):1756-1770. doi:10.1111/aogs.14206

15. Speyer LG, Marryat L, Auyeung B. Impact of COVID-19 public health safety measures on births in Scotland between March and May 2020. *Public Health*. 2022;202:76-79. doi:10.1016/j.puhe.2021.10.013

16. Chmielewska B, Barratt I, Townsend R, et al. Effects of the COVID-19 pandemic on maternal and perinatal outcomes: a systematic review and meta-analysis. *The Lancet Global Health*. 2021;9(6):e759-e772. doi:10.1016/S2214-109X(21)00079-6

17. Katikireddi SV, Lal S, Carrol ED, et al. Unequal impact of the COVID-19 crisis on minority ethnic groups: a framework for understanding and addressing inequalities. *J Epidemiol Community Health*. Published online April 21, 2021. doi:10.1136/jech-2020-216061

18. Stowe J, Smith H, Thurland K, Ramsay ME, Andrews N, Ladhani SN. Stillbirths During the COVID-19 Pandemic in England, April-June 2020. *JAMA*. 2021;325(1):86-87. doi:10.1001/jama.2020.21369

19. Riley T, Nethery E, Chung EK, Souter V. Impact of the COVID-19 pandemic on perinatal care and outcomes in the United States: An interrupted time series analysis. *Birth*. 2022;49(2):298-309. doi:10.1111/birt.12606

20. Oberndorfer M, Dundas R, Leyland AH, Pearce A. The LoCo (Lockdown Cohort)-effect: why the LoCo may have better life prospects than previous and subsequent birth cohorts. *European Journal of Public Health*. Published online May 5, 2022:ckac049. doi:10.1093/eurpub/ckac049

21. Bogin B, Varea C. COVID-19, crisis, and emotional stress: A biocultural perspective of their impact on growth and development for the next generation. *American Journal of Human Biology*. 2020;32(5):e23474. doi:10.1002/ajhb.23474

22. Luppi F, Arpino B, Rosina A. The impact of COVID-19 on fertility plans in Italy, Germany, France, Spain, and the United Kingdom. *DemRes*. 2020;43:1399-1412. doi:10.4054/DemRes.2020.43.47

23. Hale T, Angrist N, Goldszmidt R, et al. A global panel database of pandemic policies (Oxford COVID-19 Government Response Tracker). *Nat Hum Behav*. 2021;5(4):529-538. doi:10.1038/s41562-021-01079-8

24. Hedermann G, Hedley PL, Bækvad-Hansen M, et al. Danish premature birth rates during the COVID-19 lockdown. *Arch Dis Child Fetal Neonatal Ed*. 2021;106(1):93-95. doi:10.1136/archdischild-2020-319990

For peer review only - http://bmjopen.bmj.com/site/about/guidelines.xhtml

25. Casey BM, McIntire DD, Leveno KJ. The Continuing Value of the Apgar Score for the Assessment of Newborn Infants. *New England Journal of Medicine*. 2001;344(7):467-471. doi:10.1056/NEJM200102153440701

26. COVID-19 Wider Impacts – Gestation at delivery - Scottish Health and Social Care Open Data. Accessed March 18, 2022. https://www.opendata.nhs.scot/id/dataset/covid-19-wider-impacts-gestation-at-delivery

27. Cozzani M, Fallesen P, Passaretta G, Härkönen J, Bernardi F. *The Consequences of the COVID19 Pandemic for Fertility and Birth Outcomes: Evidence from Spanish Birth Registers*. SocArXiv; 2022. doi:10.31235/osf.io/f4h8e

28. Leyland AH, Ouédraogo S, Nam J, et al. Evaluation of Health in Pregnancy grants in Scotland: a natural experiment using routine data. *Public Health Research*. 2017;5(6):1-278. doi:20190814124635653

29. Rosenbaum PR. The Role of a Second Control Group in an Observational Study. *Statistical Science*. 1987;2(3):292-306. doi:10.1214/ss/1177013232

For peer review only

For peer review only - http://bmjopen.bmj.com/site/about/guidelines.xhtml

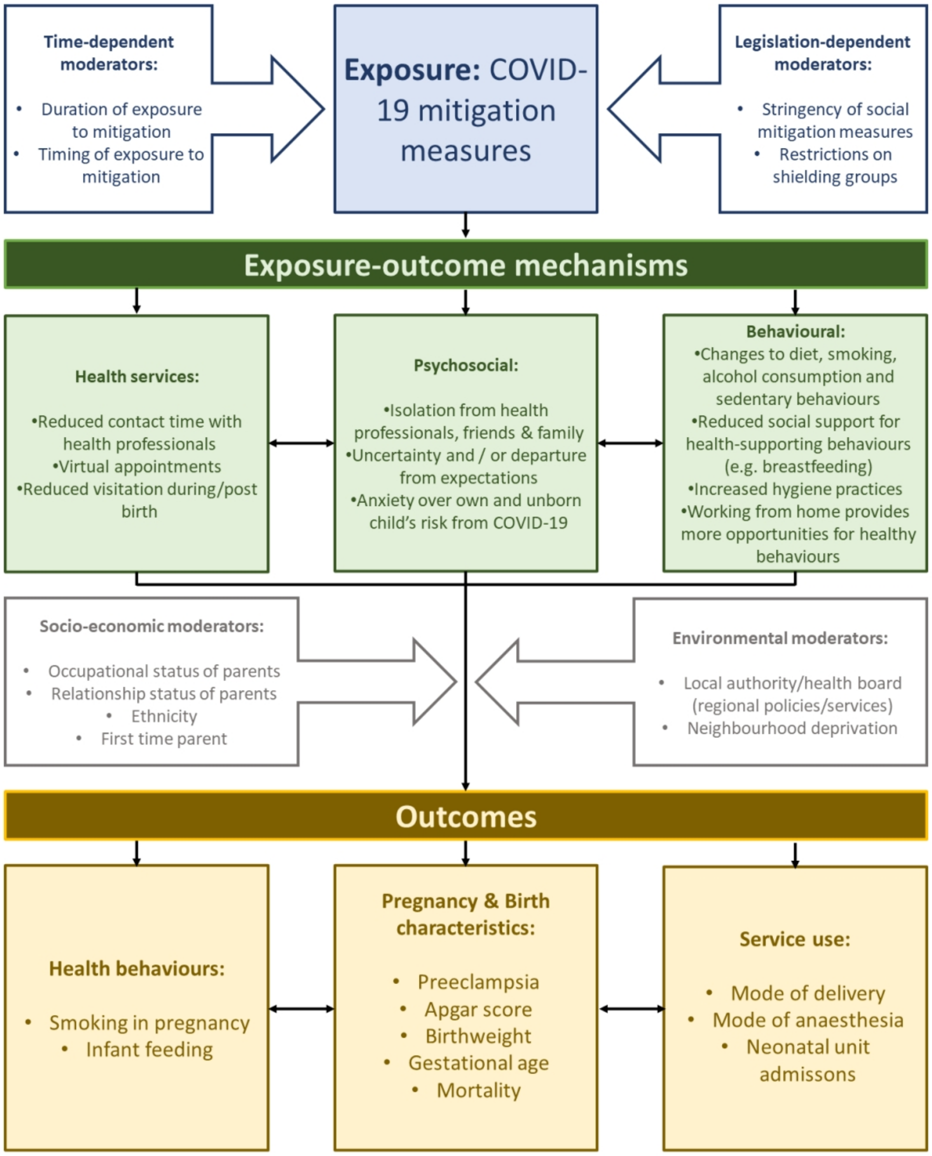

Figure 1: Logic model demonstrating the mechanisms and moderators of the wider impacts of the pandemic on pregnancy and birth outcomes

374x451mm (96 x 96 DPI)

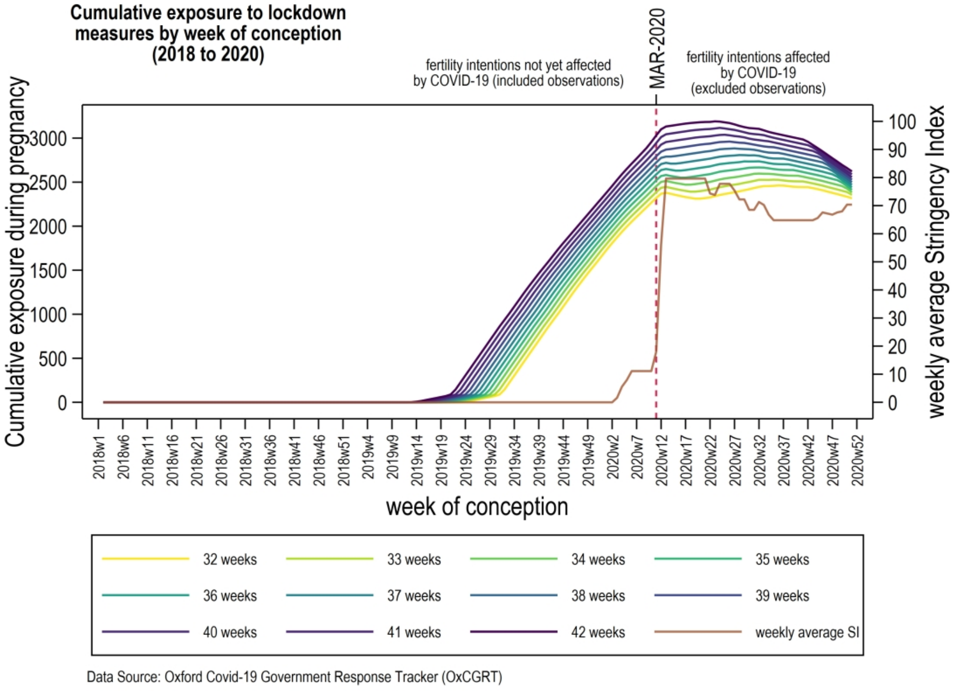

Figure 2: Visual description of our exposure variable. Sum of weekly average Stringency Index (left vertical axis) during pregnancy for each week of conception (42 weeks of gestation being the top line and 32 weeks of gestation being the bottom line) between January 2018 and December 2020. Level of cumulative exposure is shown for gestational age (32 to 42 weeks). Crude weekly average Stringency Index for Scotland is shown in brown (right vertical axis). Conceptions after March 2020 (indicated by the dashed red line) are excluded from our analyses.

1411x1058mm (72 x 72 DPI)

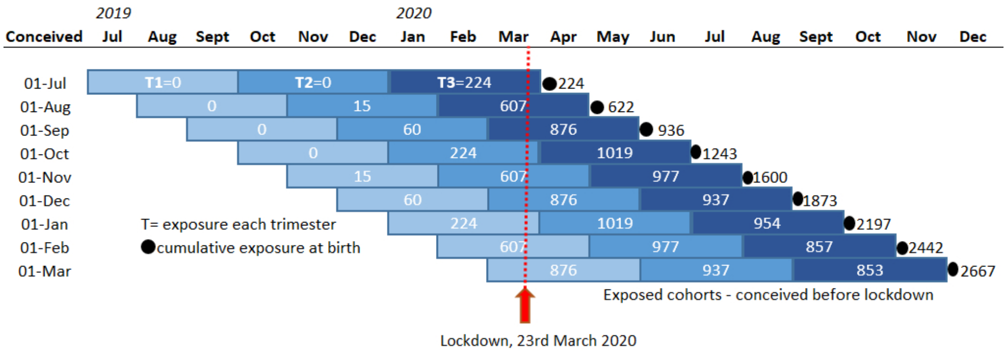

Figure 3: Exposed groups under investigation. Cumulative level of exposures presented here are the sum of weekly averages of the Stringency Index within each trimester up to month of birth. In this figure, conceptions and births are assumed to occur on the first of each month with equal gestational age. Note that, in the analyses, cumulative exposure is calculated for each mother-child pair individually and, thus, these exposure levels do not match those in Figure 2.

550x206mm (47 x 47 DPI)

The RECORD statement – checklist of items, extended from the STROBE statement, that should be reported in observational studies using routinely collected health data.

	Item No.	STROBE items	Location in manuscript where items are reported	RECORD items	Location in manuscript where items are reported
**Title and abstract**					
	1	(a) Indicate the study's design with a commonly used term in the title or the abstract (b) Provide in the abstract an informative and balanced summary of what was done and what was found	Page 1	RECORD 1.1: The type of data used should be specified in the title or abstract. When possible, the name of the databases used should be included.<br><br>RECORD 1.2: If applicable, the geographic region and timeframe within which the study took place should be reported in the title or abstract.<br><br>RECORD 1.3: If linkage between databases was conducted for the study, this should be clearly stated in the title or abstract.	Page 1 title and abstract<br><br>Page 1 abstract<br><br>Page 1 abstract
**Introduction**					
Background rationale	2	Explain the scientific background and rationale for the investigation being reported	Page 3		
Objectives	3	State specific objectives, including any prespecified hypotheses	Page 4,5<br>Figure 1		
**Methods**					
Study Design	4	Present key elements of study design early in the paper	Page 6		
Setting	5	Describe the setting, locations, and relevant dates, including periods of recruitment, exposure, follow-up, and data collection	Page 6,7,8,9		

For peer review only

Participants	6	*(a) Cohort study* - Give the eligibility criteria, and the sources and methods of selection of participants. Describe methods of follow-up<br>*Case-control study* - Give the eligibility criteria, and the sources and methods of case ascertainment and control selection. Give the rationale for the choice of cases and controls<br>*Cross-sectional study* - Give the eligibility criteria, and the sources and methods of selection of participants<br><br>*(b) Cohort study* - For matched studies, give matching criteria and number of exposed and unexposed<br>*Case-control study* - For matched studies, give matching criteria and the number of controls per case		RECORD 6.1: The methods of study population selection (such as codes or algorithms used to identify subjects) should be listed in detail. If this is not possible, an explanation should be provided.<br><br>RECORD 6.2: Any validation studies of the codes or algorithms used to select the population should be referenced. If validation was conducted for this study and not published elsewhere, detailed methods and results should be provided.<br><br>RECORD 6.3: If the study involved linkage of databases, consider use of a flow diagram or other graphical display to demonstrate the data linkage process, including the number of individuals with linked data at each stage.	Page 5, 6<br><br>Page 6,7
Variables	7	Clearly define all outcomes, exposures, predictors, potential confounders, and effect modifiers. Give diagnostic criteria, if applicable.	Page 7,8,9<br>Figure 2 and 3	RECORD 7.1: A complete list of codes and algorithms used to classify exposures, outcomes, confounders, and effect modifiers should be provided. If these cannot be reported, an explanation should be provided.	
Data sources/ measurement	8	For each variable of interest, give sources of data and details of methods of assessment (measurement).<br>Describe comparability of assessment methods if there is more than one group	Page 7,8,9		

For peer review only

Bias	9	Describe any efforts to address potential sources of bias	Page 8 to14		
Study size	10	Explain how the study size was arrived at	Page 15		
Quantitative variables	11	Explain how quantitative variables were handled in the analyses. If applicable, describe which groupings were chosen, and why	Page 7 to 13<br>Figure 2<br>Figure 3		
Statistical methods	12	(a) Describe all statistical methods, including those used to control for confounding<br>(b) Describe any methods used to examine subgroups and interactions<br>(c) Explain how missing data were addressed<br>(d) *Cohort study* - If applicable, explain how loss to follow-up was addressed<br>*Case-control study* - If applicable, explain how matching of cases and controls was addressed<br>*Cross-sectional study* - If applicable, describe analytical methods taking account of sampling strategy<br>(e) Describe any sensitivity analyses	Page 10 to 14		
Data access and cleaning methods		..		RECORD 12.1: Authors should describe the extent to which the investigators had access to the database population used to create the study population.	Page 6,7

For peer review only

				RECORD 12.2: Authors should provide information on the data cleaning methods used in the study.	
Linkage		..		RECORD 12.3: State whether the study included person-level, institutional-level, or other data linkage across two or more databases. The methods of linkage and methods of linkage quality evaluation should be provided.	Page 6,7
**Results**					
Participants	13	(a) Report the numbers of individuals at each stage of the study (*e.g.*, numbers potentially eligible, examined for eligibility, confirmed eligible, included in the study, completing follow-up, and analysed)<br>(b) Give reasons for non-participation at each stage.<br>(c) Consider use of a flow diagram	Not applicable – study protocol	RECORD 13.1: Describe in detail the selection of the persons included in the study (*i.e.,* study population selection) including filtering based on data quality, data availability and linkage. The selection of included persons can be described in the text and/or by means of the study flow diagram.	Not applicable – study protocol
Descriptive data	14	(a) Give characteristics of study participants (*e.g.*, demographic, clinical, social) and information on exposures and potential confounders<br>(b) Indicate the number of participants with missing data for each variable of interest<br>(c) *Cohort study* - summarise follow-up time (*e.g.*, average and total amount)	Not applicable – study protocol		
Outcome data	15	*Cohort study* - Report numbers of outcome events or summary measures over time<br>*Case-control study* - Report numbers in each exposure	Not applicable – study protocol		

		category, or summary measures of exposure<br>*Cross-sectional study* - Report numbers of outcome events or summary measures			
Main results	16	(a) Give unadjusted estimates and, if applicable, confounder-adjusted estimates and their precision (e.g., 95% confidence interval). Make clear which confounders were adjusted for and why they were included<br>(b) Report category boundaries when continuous variables were categorized<br>(c) If relevant, consider translating estimates of relative risk into absolute risk for a meaningful time period	Not applicable – study protocol		
Other analyses	17	Report other analyses done—e.g., analyses of subgroups and interactions, and sensitivity analyses	Not applicable – study protocol		
**Discussion**					
Key results	18	Summarise key results with reference to study objectives	Not applicable – study protocol		
Limitations	19	Discuss limitations of the study, taking into account sources of potential bias or imprecision. Discuss both direction and magnitude of any potential bias	Not applicable – study protocol	RECORD 19.1: Discuss the implications of using data that were not created or collected to answer the specific research question(s). Include discussion of misclassification bias, unmeasured confounding, missing data, and changing eligibility over time, as they pertain to the study being reported.	Not applicable – study protocol
Interpretation	20	Give a cautious overall interpretation of results considering objectives,	Not applicable – study protocol		

For peer review only - http://bmjopen.bmj.com/site/about/guidelines.xhtml

		limitations, multiplicity of analyses, results from similar studies, and other relevant evidence			
Generalisability	21	Discuss the generalisability (external validity) of the study results	Not applicable – study protocol		
**Other Information**					
Funding	22	Give the source of funding and the role of the funders for the present study and, if applicable, for the original study on which the present article is based	Page 15		
Accessibility of protocol, raw data, and programming code		Not applicable – study protocol		RECORD 22.1: Authors should provide information on how to access any supplemental information such as the study protocol, raw data, or programming code.	Not applicable – study protocol

*Reference: Benchimol EI, Smeeth L, Guttmann A, Harron K, Moher D, Petersen I, Sørensen HT, von Elm E, Langan SM, the RECORD Working Committee. The REporting of studies Conducted using Observational Routinely-collected health Data (RECORD) Statement. *PLoS Medicine* 2015; in press.

*Checklist is protected under Creative Commons Attribution (CC BY) license.

For peer review only

BMJ Open

Study Protocol: Examining the impacts of COVID-19 mitigation measures on pregnancy and birth outcomes in Scotland: A linked administrative data study

Journal:	*BMJ Open*
Manuscript ID	bmjopen-2022-066293.R1
Article Type:	Protocol
Date Submitted by the Author:	01-Dec-2022
Complete List of Authors:	Oberndorfer, Moritz; University of Glasgow MRC/CSO Social and Public Health Sciences Unit, ; Medical University of Vienna Center for Public Health<br>Henery, Paul; Public Health Scotland<br>Dundas, Ruth; University of Glasgow, MRC/CSO Social and Public Health Sciences Unit<br>Leyland, Alastair; MRC/CSO Social and Public Health Sciences Unit, University of Glasgow<br>Paranjothy, Shantini; University of Aberdeen, Health Data Science Research Centre<br>Stock, Sarah; The University of Edinburgh Usher Institute of Population Health Sciences and Informatics, Centre for Medical Informatics; Public Health Scotland,<br>Wood, Rachael; NHS National Services Scotland, Information Services Division; University of Edinburgh, Child Life and Health<br>Nelson, Scott; University of Glasgow, School of Medicine<br>Kearns, Rachel; University of Glasgow School of Medicine Dentistry and Nursing<br>Pearce, Anna; University of Glasgow MRC/CSO Social and Public Health Sciences Unit
**Primary Subject Heading**:	Public health
Secondary Subject Heading:	Obstetrics and gynaecology
Keywords:	EPIDEMIOLOGY, PUBLIC HEALTH, COVID-19, OBSTETRICS

SCHOLARONE™
Manuscripts

Study Protocol:

Examining the impacts of COVID-19 mitigation measures on pregnancy and birth outcomes in Scotland: A linked administrative data study

Authors: Moritz Oberndorfer[1,2], Paul M. Henery[3], Ruth Dundas[2], Alastair H. Leyland[2], Shantini Paranjothy[4], Sarah J. Stock[5,6], Rachael Wood[7,8], Scott M. Nelson[9], Rachel Kearns[9], Anna Pearce[2]

[1] Department of Social and Preventive Medicine, Center for Public Health, Medical University of Vienna, Vienna, Austria.

[2] MRC/CSO Social and Public Health Sciences Unit, University of Glasgow, Glasgow, UK

[3] Public Health Scotland, Edinburgh, UK

[4] Aberdeen Centre for Health Data Science, University of Aberdeen, UK

[5] The University of Edinburgh Usher Institute of Population Health Sciences and Informatics, Centre for Medical Informatics, Edinburgh, UK

[6] Public Health Scotland, Edinburgh, UK

[7] NHS National Services Scotland, Information Services Division, Edinburgh, UK

[8] Child Life and Health, University of Edinburgh, Edinburgh, UK

[9] School of Medicine Dentistry and Nursing, University of Glasgow, Glasgow, UK

Corresponding author:

Moritz Oberndorfer

Department of Social and Preventive Medicine, Center for Public Health, Medical University of Vienna

Kinderspitalgasse 15/1, 1090, Vienna, Austria

E-mail address: moritz.oberndorfer@muv.ac.at

Tel.: 0043 1 40160 34891

MRC/CSO Social and Public Health Sciences Unit, University of Glasgow

Berkeley Square, 99 Berkeley Street, Glasgow, G3 7HR, UK

E-mail address: moritz.oberndorfer@glasgow.ac.uk

Word count: ~3700

For peer review only - http://bmjopen.bmj.com/site/about/guidelines.xhtml

Abstract

Introduction

This protocol outlines aims to test the wider impacts of the COVID-19 pandemic on pregnancy and birth outcomes and inequalities in Scotland.

Method & analysis

We will analyse Scottish linked administrative data for pregnancies and births before (March 2010-March 2020) and during (April 2020-October 2020) the pandemic. The Community Health Index database will be used to link the National Records of Scotland Births and the Scottish Morbidity Record 02. The data will include about 500,000 mother-child pairs. We will investigate population-level changes in maternal behaviour (smoking at ante-natal care booking, infant feeding on discharge), pregnancy and birth outcomes (birth weight, preterm birth, Apgar score, stillbirth, neonatal death, pre-eclampsia), and service use (mode of delivery, mode of anaesthesia, neonatal unit admission) during the COVID-19 pandemic using two analytical approaches. First, we will estimate interrupted times series regression models to describe changes in outcomes comparing pre-pandemic with pandemic periods. Second, we will analyse the effect of COVID-19 mitigation measures on our outcomes in more detail by creating cumulative exposure variables for each mother-child pair using the Oxford Covid-19 Government Response Tracker. Thus, estimating a potential dose-response relationship between exposure to mitigation measures and our outcomes of interest as well as assessing if timing of exposure during pregnancy matters. Finally, we will assess inequalities in the effect of cumulative exposure to lockdown measures on outcomes using several axes of inequality: ethnicity/mother's country of birth, area deprivation (Scottish Index of Multiple Deprivation), urban-rural classification of residence, number of previous children, maternal social position (NS-SEC), and parental relationship status.

Ethics and dissemination

NHS Scotland Public Benefit and Privacy Panel for Health and Social Care (HSC-PBPP) scrutinised and approved the use of these data (1920-0097). Results of this study will be disseminated to the research community, practitioners, policy makers, and the wider public.

For peer review only - http://bmjopen.bmj.com/site/about/guidelines.xhtml

Strengths and limitations of the study

- We will use population-wide administrative data covering all mother-child pairs for children born in Scotland between March 2010 and October 2020 to study how population-level pregnancy and birth outcomes changed during the COVID-19 pandemic.
- Using the Stringency Index recorded by the Oxford Covid-19 Government Response Tracker (OxCGRT), we are able to calculate an individual level of cumulative exposure to pandemic mitigation measures for each mother-child pair in our data.
- Our effect estimates will be biased if unmeasured factors changed routine data collection (patterns of missing or misclassified data), or – for post-natal outcomes – if the characteristics of livebirths during the COVID-19 pandemic had changed in a way that is associated with our outcomes of interest.

For peer review only

For peer review only - http://bmjopen.bmj.com/site/about/guidelines.xhtml

Introduction

Early on in the Coronavirus disease 2019 (COVID-19) pandemic, concerns were raised about the widespread and unequal impacts of social mitigation measures on health and the social determinants of health[1] including for children and families[2,3]. In this protocol, we focus on parents and children during pregnancy and at birth. Figure 1 outlines three key, interlinked mechanisms through which the wider pandemic (distinct from the risks of contracting the virus) may have had negative (and sometime positive) effects on this group. The first surrounds changes to health services. Pregnant women were identified as being particularly vulnerable to the severe effects of COVID-19, prompting early advice from the NHS to adopt social distancing. This, alongside the strain put on health services by the wider pandemic, meant that the services and support for pregnant and new mothers dramatically changed.[4] Non-urgent procedures and contacts were cancelled, and resources diverted from elective to critical care. Guidance and services were quickly innovated to support new families, including the use of virtual technologies to provide health appointments, antenatal classes and hospital tours; mothers were supported to self-monitor glucose, urine and blood pressure at home; the provision of clinics in community settings increased. Partners were allowed in hospital only for the last stages of labour and no other visitors were permitted at any point during the hospital stay.[4] Although many of these restrictions have since eased, the services that young families receive have not fully returned to normal and uncertainty remains.

The second mechanism refers to psychosocial factors. Negative impacts of lockdown on mental wellbeing have been documented, alongside increases in job loss, job insecurity and universal credit claims among the adult population.[5–9] Profound changes to services and birthing plans, the disruption of feeding intentions and expectations around parenthood, and anxiety around catching the virus, have led to increased uncertainty and feelings of isolation among pregnant mothers and new families, causing psychological distress.[10,11]

Third, in the general population many health behaviours were affected, with diets becoming less healthy both in terms of quality and quantity[12] and alcohol consumption increasing, particularly among groups who were already high consumers[13]. Conversely, smoking has declined[5] and it has been hypothesised that working from home, lower exposure to air pollutants, and better hygiene habits may have benefited foetal development and health[14]. Hospital support for breastfeeding immediately after birth has remained[10], and breastfeeding rates upon discharge have not necessarily been affected[15]. However, lack of support from friends and family, mother and baby groups, and health professionals has been highlighted as a barrier to feeding after returning home.[10]

For peer review only - http://bmjopen.bmj.com/site/about/guidelines.xhtml

Our aim is not to test these different mechanisms, but to first establish the overall impacts of the pandemic on various mother and infant outcomes, and inequalities in these outcomes, in Scotland. This will provide a better understanding of potential future health challenges and to inform responses to the ongoing and any future pandemics. A comprehensive investigation of pregnancy and birth outcomes, in Scotland during March-May 2020 (compared to two years previous), found that some procedural outcomes showed changes in the expected direction (e.g. length of hospital stay decreased), but few changes in maternal and infant health outcomes.[15] Few signs of negative impacts (in high income countries) have also been detected in international systematic reviews and meta-analyses[14,16], with the exception of maternal mental health[16]. However, while the overall picture is positive, it remains plausible that these studies have overlooked differential effects occurring at the sub-group level. In the case of the three of proposed mechanisms discussed above, it is likely that some groups, including those from less advantaged social circumstances, first time mothers, and ethnic minority, groups have fared worse than others.[1,17] There are also some indications in the limited evidence base that birth and pregnancy have worsened from some groups and not others. For example, there was no change in stillbirths in England overall, but rates had increased in North England.[18] In the United States, new-born readmission rates among first time mothers were higher after the pandemic, while multiparous women were less likely to experience pre-term birth rates, low Apgar scores and hospital readmissions.[19] Furthermore, it is possible that early studies considering outcomes only at the very start of the pandemic may have overlooked impacts on expectant mothers who were exposed to social mitigation measures for longer durations of pregnancy.

We aim to estimate the wider impacts of the COVID-19 pandemic on pregnancy and birth outcomes and inequalities in Scotland. More specifically, we aim to estimate changes in health and pregnancy outcomes as a result of the pandemic. We will take a natural experiment approach to identify any step change trends in outcomes at the start of the pandemic, limiting our analyses to pregnancies which were conceived before the pandemic, to avoid introducing bias due to the changing socio-demographic characteristics of conceptions which occurred after the start of the pandemic[20,21]. As part of this aim, we will investigate whether exposure to mitigation measures had a differential effect on our outcomes across several axes of inequalities. Second, we aim to consider the cumulative effects of social mitigation measures across pregnancy. To this end we will use the stringency index (which measures the strictness of policies that primarily restrict people's behaviour) and compare cohorts with different lengths or intensity of exposure. Additionally, we aim to consider timing of exposure, as it is possible that, for some outcomes, any impacts of the stresses related to the pandemic and social mitigation measures might be greater during some trimesters of pregnancy than others[22].

For peer review only - http://bmjopen.bmj.com/site/about/guidelines.xhtml

Methods

Patient and public involvement

This secondary analysis of data will not directly involve the public or patients. Findings will be disseminated to relevant health professionals and interest groups to maximise benefits for service provision throughout Scotland.

Study design and population

Our study population includes live births born between March 2010 and October 2020. More precisely, our population of interest consists of live births conceived before the pandemic who have not been exposed to COVID-19 mitigation measures in utero (live births between March 2010 and February 2020) and those who were conceived before the pandemic but were exposed to mitigation measures in utero (live births between March 2020 and October 2020).

We will employ two analytical approaches, each informed by the logic model in Figure 1. In our first analytical approach, we will provide, using interrupted time series regression models, a descriptive visualisation of how outcome variables changed between pre-pandemic (March 2010-February 2020) and pandemic (March 2020-October 2020). Births from November 2020 onwards will be excluded from our regression analysis since the majority were conceived during lockdown, and the pandemic and its socioeconomic consequences might have affected fertility and thereby the characteristics of new families in ways that we cannot fully account for.[20,21,23] In this first approach we will ignore variation in exposure to mitigation measures during pregnancy and at birth as we aim to estimate the average population level impact of COVID-19 mitigation measures on pregnancy and birth outcomes.

In our second analytical approach, we will investigate the relationship between the outcomes and exposure to mitigation measures in more detail. As the intensity, duration, and timing of exposure to COVID-19 mitigation measures is dependent on the date of conception and duration of pregnancy, each mother and child pair will be given an individually calculated level of cumulative exposure to mitigation measures in Scotland using the Stringency Index created by the Oxford Covid-19 Government Response Tracker (OxCGRT)[24]. This allows us to estimate a potential dose-response relationship between exposure to mitigation measures as well as potential effect moderation by timing of exposure (focusing on trimesters).

For peer review only - http://bmjopen.bmj.com/site/about/guidelines.xhtml

Databases

We will use linked data from the below datasets:

National Records of Scotland (NRS) Births: The NRS holds information on all births registered in Scotland since 1975. These records include information on date and location of the birth and details of the registered parent(s), including their marital/relationship status and their occupational status.

Scottish Morbidity Record 02 (SMR02): SMR02 records all maternity and infant inpatient and day case episodes in Scotland. Around 50% episodes relate to births and it was these records that were requested for the purposes of the cohort. These include demographic characteristics and information relating to the birth and clinical management.

National Records Scotland (NRS) and The Scottish Stillbirths and Infant Deaths Survey (SSBIDS): register of all births, stillbirths, and infant (including neonatal) deaths.

Scottish Birth Records (SBR): all records of a baby's neonatal care in Scotland

Community Health Index (CHI) Database: This contains a unique identifier for all NHS users in Scotland (~99% of population) and is used to link the above datasets.

Outcomes

We chose outcomes that could feasibly be affected by social mitigation measures (Figure 1 logic model) and for their relevance for subsequent child and adult health. We grouped them into maternal behaviours, birth and pregnancy outcomes, and service use.

Maternal behaviours: Smoking in pregnancy, usually measured during the ante-natal care booking (~8 -12 weeks of pregnancy) supplemented by information collected at any subsequent ante-natal appointments (yes; no). Infant feeding at discharge from hospital (breastfeeding - yes; no).

Birth and pregnancy characteristcs: Birthweight in grams (continuous variable); low birthweight <2500g and high birthweight >4000g. Similarly, gestational age will be considered as a continuous variable and categorised to identify preterm birth (delivery before 37^{+0} weeks of gestation) and late gestational age (≥42^{+0} weeks). We will carry out sensitivity analyses differentiating different degrees of prematurity (extremely preterm: <28^{+0} weeks; very preterm: 28^{+0} to 31^{+6} weeks; moderate to late preterm: 32^{+0} to 36^{+6} weeks) and low birthweight (extremely low: <1000g; very low: 1000 to 1499g; low: 1500g to 2499g), since previous research has found delays in extreme prematurity which only manifest in reductions in 'very premature'[25]. Additionally, we will analyse birthweight standardised for gestational age and consequently small for gestational age (SGA) as well as large for gestational age

For peer review only - http://bmjopen.bmj.com/site/about/guidelines.xhtml

(LGA) as outcomes to explicitly focus on fetal growth. The Apgar score, measured within the first five minutes after delivery, assesses five characteristics (heart rate, respiratory effort, muscle tone, reflex irritability, colour), and can be dichotomised to measure good to excellent infant health (score of 7 or higher[26]). Additionally, we will examine hypertensive disease of pregnancy by combining ICD10 codes for gestational hypertension and pre-eclampsia. We will not examine these outcomes separately as they are clinically closely linked and allocation to ICD10 codes may vary in precision across areas. Lastly, we will explore pandemic-induced changes in the prevalence of gestational diabetes. However, this outcome is likely affected via changes in the uptake of screening and testing for gestational diabetes during the COVID-19 pandemic.

Health services use: Mode of delivery will consist of four categories (spontaneous vaginal, assisted vaginal, planned caesarean, emergency caesarean), mode of anaesthesia (spinal, general anaesthesia, epidural), and neonatal unit admissions.

Most of the outcomes under examination are relatively common (e.g., rate of preterm births is 65 per 1,000). The least common are stillbirths (5 per 1,000) and low birth weight (20 per 1,000). With 27,100 births occurred during the pandemic period (April 2020-October 2020[27]), these outcomes are relatively infrequent.

Secondary outcomes: We will also consider changes before/during the pandemic in the following secondary outcomes: miscarriage (loss of baby during first 23 weeks of pregnancy), stillbirths (loss of a baby after 24 weeks of gestation) and neonatal deaths (first 28 days after delivery). Some of these outcomes are very rare (e.g. neonatal deaths is <0.2%) and so may only be used to identify bias, with outcome data not reported. Analysis of changes in our secondary outcomes will inform our analysis of post-natal outcomes. If, for example, rates of stillbirths and miscarriages were higher during the pandemic compared to pre-pandemic periods, we expect the pandemic to have an indirect protective effect on post-natal outcomes via this selection mechanism.

Exposure

For our first approach – the interrupted time series analysis - we will use dummy variables to indicate whether the outcome (measured at booking or at birth, depending on the outcome) was observed during pre-pandemic (before first lockdown measures in March 2020) or pandemic periods (April 2020 to October 2020).

For our second analysis, we will calculate an individual level of cumulative exposure for each mother-child pair using the OxCGRT. The OxCGRT has recorded government responses to the COVID-19 pandemic. Methodological details of the OxCGRT have been described elsewhere.[24] As a measure of the stringency of lockdown measures, we will use the OxCGRT Stringency Index (SI) which comprises 9

For peer review only - http://bmjopen.bmj.com/site/about/guidelines.xhtml

different indicators (school closing, workplace closing, cancel public events, restriction on gathering size, close public transport, stay at-home order requirements, restrictions on internal movement, restriction on international travel, public health campaigns). The SI ranges from 0 to 100 and has been recorded daily since January 2020. For Scotland, the SI increased drastically between the first week of March 2020 (SI=11.11) to the highest value during our observation period in the last week of March 2020 (SI=79.63). The time series of weekly average SI is shown in figure 2 (right y-axis). The COVID-19 strategy of the Scottish government can be found at https://www.gov.scot/collections/coronavirus-covid-19-strategic-approach/ .

Cumulative exposure to lockdown measures will be calculated by the sum of weekly averages of SI during pregnancy and up until the occurrence of the outcome. Figure 2 visualises the level of cumulative exposure for mother-child pairs by week of conception for different gestational ages. As raised in the introduction, it is possible that timing of exposure to social mitigation matters. We will therefore also examine cumulative exposure within each trimester of pregnancy.

Population characteristics and confounding factors

All models will include dummy variables indicating which month the outcomes were observed (with January being the reference) to account for seasonality and the correlation between month of birth and cumulative exposure.

In the second analytic approach, an association between our cumulative exposure variable and duration of pregnancy arises automatically as mothers with the same conception date but different pregnancy durations will have been exposed to different levels of cumulative exposure at delivery. Therefore, duration of pregnancy will be correlated with the cumulative exposure to SI of a mother-child pair and a post-natal outcome (e.g., birthweight) of interest and thus needs to be adjusted for.

Yet, duration of pregnancy is not only a confounder of the exposure-outcome relationship for post-natal outcomes (because it has a deterministic relationship with our cumulative exposure) but may also be a mediator. Exposure to the pandemic might affect gestational age (e.g., by changing maternal behaviour or health services) which in turn affects post-natal outcomes (birthweight, Apgar score, neonatal death, infant feeding on discharge, mode of delivery, mode of anaesthesia, neonatal unit admission). Through adjusting for gestational age, we will therefore remove confounding effects but potentially block part of the effect of interest if it is also a mediator. Analyses on gestational age as an outcome will inform the extent of this potential overadjustment for post-natal outcomes.

Change in incidence of miscarriage, pregnancy terminations, stillbirths, and maternal emigration behaviour during pregnancy due to COVID-19 mitigation measures may also act as potential mediators of the exposure-outcome relationship. Because the pandemic might have increased the likelihood of

For peer review only - http://bmjopen.bmj.com/site/about/guidelines.xhtml

these events, this pathway could potentially result in a protective effect of the exposure on post-natal outcomes (for example birthweight). Blocking these mediating pathways from exposure to outcome will avoid potentially counteracting, more proximate causes of the association between SI and post-natal outcomes that might deceptively lead to attenuated effects ('live birth bias'). This will be partially achieved by the control variables introduced in Model (3), as we expect these characteristics of mother-child pairs (maternal age, sex of baby, maternal NS-SEC, SIMD, and urban-rural classification of residence) to be associated with a potential change in likelihood of these events due to the pandemic. Thus, the resulting estimand is the average total effect of our exposure on post-natal outcomes controlled for potential in utero selection effects. It is not an aim of the study to examine other mediating mechanisms.

We will also adjust for variables that are associated with the outcome but not with the exposure – to take account of potential time trends in outcomes, including, where sufficiently complete: maternal age, maternal occupational class measured by NS-SEC, ethnicity of mother, sex of the baby, Scottish Index of Multiple Deprivation, and urban-rural classification of residence. Informed by previous work[28], we expect a large proportion of missing information on maternal ethnicity (around 50%) but high completeness (>90%) in the other variables.

Impacts on inequalities

In both approaches, several axes of inequality will be examined to consider whether the impacts of the pandemic have been differential: ethnicity/mother's country of birth (depending on completeness and available sample size), area deprivation (Scottish Index of Multiple Deprivation (SIMD)), urban-rurality classification of residence, first time mothers, maternal social position (National Statistics Socioeconomic Classification (NS-SEC)), and relationship status of parents (sole registrations, separated, cohabitating, married). We will measure both absolute and relative inequalities.

Relationship status, SIMD, and urban-rural classification of residence can possibly change due to COVID-19 mitigation measures. Using our first analytical approach, we will assess potential step or slope changes in the number of births born to mothers in different relationship, SIMD, and urban/rural categories following March 2020. As we expect no compositional changes due to selection into pregnancy within our chosen observation period, this analysis will inform to which extent compositional change regarding area level characteristics (SIMD and urban/rural classification) were due to maternal moving behaviour.

Statistical analysis

In the first approach, we will use interrupted time series (ITS) regression models to describe time trends in the outcomes. Therefore, we will constrain this analysis to linear functions of time. Covariates

For peer review only - http://bmjopen.bmj.com/site/about/guidelines.xhtml

in these models will be time (weeks, or months) since first date of collected data, a dummy variable indicating whether an observation belongs to the exposed or unexposed group, an interaction between time and the exposure dummy variable, and dummy variables indicating in which month the outcome was observed with January being the reference month. Our data is structured by two levels: mother-child pairs nested within small geographic areas. Therefore, we will use multilevel modelling throughout our regression analysis. Model (1) exemplarily shows the formal specification for the continuous outcome birth weight y_{ij} of mother-child pair *i* nested within small area (data zone) *j*. For non-continuous outcomes (smoking, infant feeding, LBA, HBW, prematurity, SGA, LGA, method of delivery, mode of anaesthesia, preeclampsia, neonatal admissions, stillbirth, neonatal death), we will use weekly prevalence rate (number of weekly events/number of weekly live births). For the least common outcomes (stillbirth and LBW), we will use monthly prevalence rates if necessary.

$$y_{ij} = \beta_0 + \beta_1 week_{ij} + \beta_2 exposed_{ij} + \beta_3 week_{ij} X exposed_{ij} + \sum_{t=1}^{11} \beta_t month_{ij} + u_{0j} + \varepsilon_{0ij}, \quad u_{0j} \sim N(0, \sigma_{u0}^2),\ \varepsilon_{0ij} \sim N(0, \sigma_{e0}^2) \quad (1)$$

In the second approach, the exposure is the cumulative Stringency Index and we will adjust for potential confounders. As an example, we formally describe our models for the continuous outcome birthweight below.

$$y_{ij} = \beta_0 + \beta_1 SI_{ij} + \beta_2 DoP_{ij} + \sum_{t=1}^{11} \beta_t month_{ij} + u_{0j} + \varepsilon_{0ij}, \quad u_{0j} \sim N(0, \sigma_{u0}^2),\ \varepsilon_{0ij} \sim N(0, \sigma_{e0}^2) \quad (2)$$

Model (2) presents our most parsimonious model specification, wherein y_{ij} is birthweight (in grams) measured for mother-child pair *i* in data zone *j*, SI_{ij} is the sum of weekly average Stringency Index during pregnancy of mother-child pair *ij*, DoP_{ij} is the duration of pregnancy (in weeks) for mother-child pair *ij*, and $month_{ij}$ is a dummy variable that indicates in which month birth was given with January being the reference category. In Model (3), we further include the neutral control variables maternal age, sex of baby, maternal NS-SEC, SIMD, and urban-rural classification of residence. In case there is considerable missing information in a neutral control variable, we will omit it from our models as the risk of bias induced by missing not at random likely outweighs the potential gains of a neutral control.

$$y_{ij} = \beta_0 + \beta_1 SI_{ij} + \beta_2 DoP_{ij} + \sum_{t=1}^{11} \beta_t month_{ij} + \beta_3 age_{ij} + \beta_4 sex_{ij} + \beta_5 NSSEC_{ij} + \beta_6 SIMD_{ij} + \beta_7 urban_{ij} + u_{0j} + \varepsilon_{0ij}, \quad u_{0j} \sim N(0, \sigma_{u0}^2),\ \varepsilon_{0ij} \sim N(0, \sigma_{e0}^2) \quad (3)$$

Moreover, for post-natal outcomes, we will explore whether timing of exposure matters by including variables for cumulative exposure during each trimester of pregnancy as shown in Model (4). Wherein $SI1_{ij}$, $SI2_{ij}$, $SI3_{ij}$ is the sum of weekly average Stringency Index during the first, second, and third

For peer review only - http://bmjopen.bmj.com/site/about/guidelines.xhtml

trimester of pregnancy of mother-child pair *ij.* Figure 3 illustrates cumulative exposure to mitigation measures during each trimester. As our data do not include cohorts that experienced high levels of exposure during their first trimester and low exposure during their third trimester, we will only test differences in the effect of exposure during the third and second trimester[22].

$$y_{ij} = \beta_0 + \beta_1 SI1_{ij} + \beta_2 SI2_{ij} + \beta_3 SI3_{ij} + \beta_4 DoP_{ij} + \sum_{t=1}^{11} \beta_t month_{ij} + \beta_5 age_{ij} + \beta_6 sex_{ij} + \beta_7 NSSEC_{ij} + \beta_8 SIMD_{ij} + \beta_9 urban_{ij} + u_{0j} + \varepsilon_{0ij}, \;\; u_{0j} \sim N\left(0,\sigma^2_{u0}\right), \;\; \varepsilon_{0ij} \sim N\left(0,\sigma^2_{e0}\right) \quad (4)$$

To help with the interpretation of our results, we will present estimated values for hypothetical plausible levels of cumulative exposure (i.e. exposure to 3,6,9 months of a Stringency Index of 50,60,70, etc.). In addition, we will present the estimated average outcome values at specific time points. Note that no mother-child pair in our data has experienced a different level of exposure at the same date of birth and length of gestation.

We will examine differential effects by including interaction terms with the modifying variables (see inequalities section). Where interactions appear to be meaningful, we will stratify the models. Inequalities in effect sizes will be examined by comparing average effects between levels of moderating variables. In Model (5), we exemplarily show the specification of such a model for inequalities in the effect of our exposure variable along parental NS-SEC.

$$y_{ij} = \beta_0 + \beta_1 SI_{ij} + \beta_2 DoP_{ij} + \sum_{t=1}^{11} \beta_t month_{ij} + \beta_3 age_{ij} + \beta_4 sex_{ij} + \beta_5 NSSEC_{ij} + \beta_6 SIMD_{ij} + \beta_7 urban_{ij} + \beta_8 \, SI_{ij} \, x \, NSSEC_{ij} + u_{0j} + \varepsilon_{0ij}, \;\; u_{0j} \sim N\left(0,\sigma^2_{u0}\right), \;\; \varepsilon_{0ij} \sim N\left(0,\sigma^2_{e0}\right) \quad (5)$$

Multilevel linear models will be used for continuous outcomes (as appropriate for the distribution of outcome data), with multilevel binary and multinomial logistic regression models used for binary and categorical outcomes respectively. All models will be estimated by maximum likelihood. We will derive prevalence ratios and absolute differences from model estimates.

Sensitivity analysis

In our ITS regression analysis, the included linear time trend and month indicator variables may not fully address the autocorrelation of observations. We will therefore inspect the autocorrelation function and partial autocorrelation function of our model residuals and resort to (seasonal) Autoregressive Integrated Moving Average (SARIMA) Models if necessary.

We will explore non-linearity in the effect of cumulative exposure to lockdown measures by re-estimating our models with a quadratic functional form of the exposure variable as well as a semi-parametric specification, in which we use quintiles of the exposure variable as cut-offs to form discrete

For peer review only - http://bmjopen.bmj.com/site/about/guidelines.xhtml

levels of cumulative exposure. We will repeat analyses limited to singleton births. Additionally, we will analyse induced and spontaneous preterm births separately (if sample size is sufficient). Depending on the partnership status of parents at birth registration, we will also have information on paternal NS-SEC. We will conduct sensitivity analyses in which we exchange maternal with paternal NS-SEC where available, as well as taking the higher occupational class in the household.

As noted previously, excluding births conceived during lockdowns will reduce unmeasured or residual confounding due to changed sociodemographic parental characteristics likely associated with the outcomes.[20,21] However, changes in the likelihood of miscarriage, pregnancy terminations, stillbirths, neonatal deaths and maternal emigration behaviour during pregnancy may still introduce bias for post-natal outcomes. We will explore this by analysing time trends for available variables (stillbirth, miscarriage, neonatal death) using interrupted time series regression as described above. If this analysis suggests that our exposure-outcome relationship is susceptible to such potential selection bias, we will further control (where possible) for variables that are likely associated with miscarriage, pregnancy terminations, stillbirths, and maternal emigration behaviour during pregnancy as well as the outcomes (but not affected by the exposure).

Finally, we will explore unmeasured confounding by splitting our data in multiple unexposed comparison groups (April to October for each year between 2010 and 2019).[29] Systematic differences in our outcomes between unexposed groups conditional on the covariates listed above will be tested by estimating the effect of dummy variables indicative of which comparison group a mother-child pair belongs to using regression analyses. Systematic differences in the outcomes between unexposed comparison groups even after adjusting for our set of covariates will reveal whether there is potential unmeasured confounding in respect to the effect of our cumulative exposure variable on the outcomes. The exposure-outcome relationship will then be estimated using varying sets of unexposed comparison groups against the exposed group of mother-child pairs (April to October 2020). Resulting effect sizes will be shown in forest plots and a pooled effect will be estimated by random-effects meta-analysis. In case unexposed comparison groups indeed differ in respect to our outcomes after covariate adjustment, results of this pooled analysis will be interpreted in light of unexplainable differences between unexposed groups.

Sample size

Our sample consists of all child and mother pairs for children born in Scotland between March 2010 and October 2020. Sample size is expected to be *n*~500,000 mother-child pairs (estimated based on an average of 50,000 births per annum).

Missing data

For peer review only - http://bmjopen.bmj.com/site/about/guidelines.xhtml

We will document levels of missing data in all variables of interest, over time and according to the potential effect moderators, for two reasons. First, understanding how data collection was impacted during the early stages of the pandemic can inform responses to future pandemics. Second, changes in patterns of missingness in the data, due to the pandemic, could introduce bias. In case of considerable levels of missing data, item missingness will be addressed using multiple imputation by chained equations.

Ethics and dissemination

Use of the data have been approved by the Public Benefit and Privacy Panel for Health and Social Care. Results of this research will be disseminated in peer reviewed presentations at public health national and international conferences and open access, peer reviewed journal articles. We will produce a briefing paper for policy-makers and practitioners and will work with in-house press advisors to ensure visibility in newspapers, radio etc. and on our COVID-19 Unit webpage.

Authors' contributions

RD, AHL, MO and AP conceived the study. SMN obtained the data and associated approvals. PMH, SP, SJS, RW, SMN, RK contributed to the conception of the study design. MO, AP and PMH drafted the study protocol. RD, AHL, PMH, SP, SJS, RW, SMN, RK provided critical feedback on the draft manuscript and approved the final version.

Competing interests statement

The authors declare that they have no competing interest.

Funding statement

The work of the authors was supported by the Medical Research Council (MC_UU_00022/2) and the Scottish Government Chief Scientist Office (SPHSU17). AP also receives support from the Wellcome Trust (205412/Z/16/Z). MO is also supported by the Marietta-Blau Scholarship (MPC-2021-00178; funded by the Austrian Federal Ministry of Education, Science and Research). AHL, AP, and RD are members of, and receive support from, the UK Prevention Research Partnership Maternal and Child Health Network (MR/S037608/1).

Data sharing statement

Linked administrative data used for this study cannot be shared by the authors. The Stringency Index is openly available (https://ourworldindata.org/covid-stringency-index).

For peer review only - http://bmjopen.bmj.com/site/about/guidelines.xhtml

Word Count

~4000

Figure captions

Figure 1: *Logic model demonstrating the mechanisms and moderators of the wider impacts of the pandemic on pregnancy and birth outcomes*

Figure 2: *Visual description of our exposure variable. Sum of weekly average Stringency Index (left vertical axis) during pregnancy for each week of conception (42 weeks of gestation being the top line and 32 weeks of gestation being the bottom line) between January 2018 and December 2020. Level of cumulative exposure is shown for gestational age (32 to 42 weeks). Crude weekly average Stringency Index for Scotland is shown in brown (right vertical axis). Conceptions after March 2020 (indicated by the dashed red line) are excluded from our analyses.*

Figure 3: *Exposed groups under investigation. Cumulative level of exposures presented here are the sum of weekly averages of the Stringency Index within each trimester up to month of birth. In this figure, conceptions and births are assumed to occur on the first of each month with equal gestational age. Note that, in the analyses, cumulative exposure is calculated for each mother-child pair individually and, thus, these exposure levels do not match those in Figure 2.*

Ethics Approval:

NHS Scotland Public Benefit and Privacy Panel for Health and Social Care (HSC-PBPP) scrutinised and approved the use of these data (1920-0097).

References

1. Bambra C, Riordan R, Ford J, Matthews F. The COVID-19 pandemic and health inequalities. *J Epidemiol Community Health*. Published online June 12, 2020. doi:10.1136/jech-2020-214401

2. Hefferon C, Taylor C, Bennett D, et al. Priorities for the child public health response to the COVID-19 pandemic recovery in England. *Archives of Disease in Childhood*. 2021;106(6):533-538. doi:10.1136/archdischild-2020-320214

3. Goldfeld S, O'Connor E, Sung V, et al. Potential indirect impacts of the COVID-19 pandemic on children: a narrative review using a community child health lens. *Med J Aust*. 2022;216(7). doi:10.5694/mja2.51368

4. Jardine J, Relph S, Magee L, et al. Maternity services in the UK during the coronavirus disease 2019 pandemic: a national survey of modifications to standard care. *BJOG: An International Journal of Obstetrics & Gynaecology*. 2021;128(5):880-889. doi:10.1111/1471-0528.16547

5. Niedzwiedz CL, Green MJ, Benzeval M, et al. Mental health and health behaviours before and during the initial phase of the COVID-19 lockdown: longitudinal analyses of the UK Household Longitudinal Study. *J Epidemiol Community Health*. 2021;75(3):224-231. doi:10.1136/jech-2020-215060

6. Oberndorfer M, Dorner TE, Brunnmayr M, Berger K, Dugandzic B, Bach M. Health-related and socio-economic burden of the COVID-19 pandemic in Vienna. *Health & Social Care in the Community*. 2021;n/a(n/a). doi:10.1111/hsc.13485

7. Serrano-Alarcón M, Kentikelenis A, Mckee M, Stuckler D. Impact of COVID-19 lockdowns on mental health: Evidence from a quasi-natural experiment in England and Scotland. *Health Econ*. Published online November 12, 2021:10.1002/hec.4453. doi:10.1002/hec.4453

For peer review only - http://bmjopen.bmj.com/site/about/guidelines.xhtml

For peer review only

8. Chandola T, Kumari M, Booker CL, Benzeval M. The mental health impact of COVID-19 and lockdown-related stressors among adults in the UK. *Psychol Med*. Published online December 7, 2020:1-10. doi:10.1017/S0033291720005048

9. Patel K, Robertson E, Kwong ASF, et al. Psychological Distress Before and During the COVID-19 Pandemic Among Adults in the United Kingdom Based on Coordinated Analyses of 11 Longitudinal Studies. *JAMA Network Open*. 2022;5(4):e227629. doi:10.1001/jamanetworkopen.2022.7629

10. Vazquez-Vazquez A, Dib S, Rougeaux E, Wells JC, Fewtrell MS. The impact of the Covid-19 lockdown on the experiences and feeding practices of new mothers in the UK: Preliminary data from the COVID-19 New Mum Study. *Appetite*. 2021;156:104985. doi:10.1016/j.appet.2020.104985

11. Fallon V, Davies SM, Silverio SA, Jackson L, De Pascalis L, Harrold JA. Psychosocial experiences of postnatal women during the COVID-19 pandemic. A UK-wide study of prevalence rates and risk factors for clinically relevant depression and anxiety. *J Psychiatr Res*. 2021;136:157-166. doi:10.1016/j.jpsychires.2021.01.048

12. McBride E, Arden MA, Chater A, Chilcot J. The impact of COVID-19 on health behaviour, well-being, and long-term physical health. *Br J Health Psychol*. 2021;26(2):259-270. doi:10.1111/bjhp.12520

13. Public Health England. Monitoring alcohol consumption and harm during the COVID-19 pandemic. Published online 2021.

14. Yang J, D'Souza R, Kharrat A, et al. COVID-19 pandemic and population-level pregnancy and neonatal outcomes: a living systematic review and meta-analysis. *Acta Obstet Gynecol Scand*. 2021;100(10):1756-1770. doi:10.1111/aogs.14206

15. Speyer LG, Marryat L, Auyeung B. Impact of COVID-19 public health safety measures on births in Scotland between March and May 2020. *Public Health*. 2022;202:76-79. doi:10.1016/j.puhe.2021.10.013

16. Chmielewska B, Barratt I, Townsend R, et al. Effects of the COVID-19 pandemic on maternal and perinatal outcomes: a systematic review and meta-analysis. *The Lancet Global Health*. 2021;9(6):e759-e772. doi:10.1016/S2214-109X(21)00079-6

17. Katikireddi SV, Lal S, Carrol ED, et al. Unequal impact of the COVID-19 crisis on minority ethnic groups: a framework for understanding and addressing inequalities. *J Epidemiol Community Health*. Published online April 21, 2021. doi:10.1136/jech-2020-216061

18. Stowe J, Smith H, Thurland K, Ramsay ME, Andrews N, Ladhani SN. Stillbirths During the COVID-19 Pandemic in England, April-June 2020. *JAMA*. 2021;325(1):86-87. doi:10.1001/jama.2020.21369

19. Riley T, Nethery E, Chung EK, Souter V. Impact of the COVID-19 pandemic on perinatal care and outcomes in the United States: An interrupted time series analysis. *Birth*. 2022;49(2):298-309. doi:10.1111/birt.12606

20. Oberndorfer M, Dundas R, Leyland AH, Pearce A. The LoCo (Lockdown Cohort)-effect: why the LoCo may have better life prospects than previous and subsequent birth cohorts. *European Journal of Public Health*. Published online May 5, 2022:ckac049. doi:10.1093/eurpub/ckac049

21. Cozzani M, Fallesen P, Passaretta G, Härkönen J, Bernardi F. *The Consequences of the COVID19 Pandemic for Fertility and Birth Outcomes: Evidence from Spanish Birth Registers*. SocArXiv; 2022. doi:10.31235/osf.io/f4h8e

22. Bogin B, Varea C. COVID-19, crisis, and emotional stress: A biocultural perspective of their impact on growth and development for the next generation. *American Journal of Human Biology*. 2020;32(5):e23474. doi:10.1002/ajhb.23474

23. Luppi F, Arpino B, Rosina A. The impact of COVID-19 on fertility plans in Italy, Germany, France, Spain, and the United Kingdom. *DemRes*. 2020;43:1399-1412. doi:10.4054/DemRes.2020.43.47

For peer review only - http://bmjopen.bmj.com/site/about/guidelines.xhtml

24. Hale T, Angrist N, Goldszmidt R, et al. A global panel database of pandemic policies (Oxford COVID-19 Government Response Tracker). *Nat Hum Behav*. 2021;5(4):529-538. doi:10.1038/s41562-021-01079-8

25. Hedermann G, Hedley PL, Bækvad-Hansen M, et al. Danish premature birth rates during the COVID-19 lockdown. *Arch Dis Child Fetal Neonatal Ed*. 2021;106(1):93-95. doi:10.1136/archdischild-2020-319990

26. Casey BM, McIntire DD, Leveno KJ. The Continuing Value of the Apgar Score for the Assessment of Newborn Infants. *New England Journal of Medicine*. 2001;344(7):467-471. doi:10.1056/NEJM200102153440701

27. COVID-19 Wider Impacts – Gestation at delivery - Scottish Health and Social Care Open Data. Accessed March 18, 2022. https://www.opendata.nhs.scot/id/dataset/covid-19-wider-impacts-gestation-at-delivery

28. Leyland AH, Ouédraogo S, Nam J, et al. Evaluation of Health in Pregnancy grants in Scotland: a natural experiment using routine data. *Public Health Research*. 2017;5(6):1-278. doi:20190814124635653

29. Rosenbaum PR. The Role of a Second Control Group in an Observational Study. *Statistical Science*. 1987;2(3):292-306. doi:10.1214/ss/1177013232

For peer review only

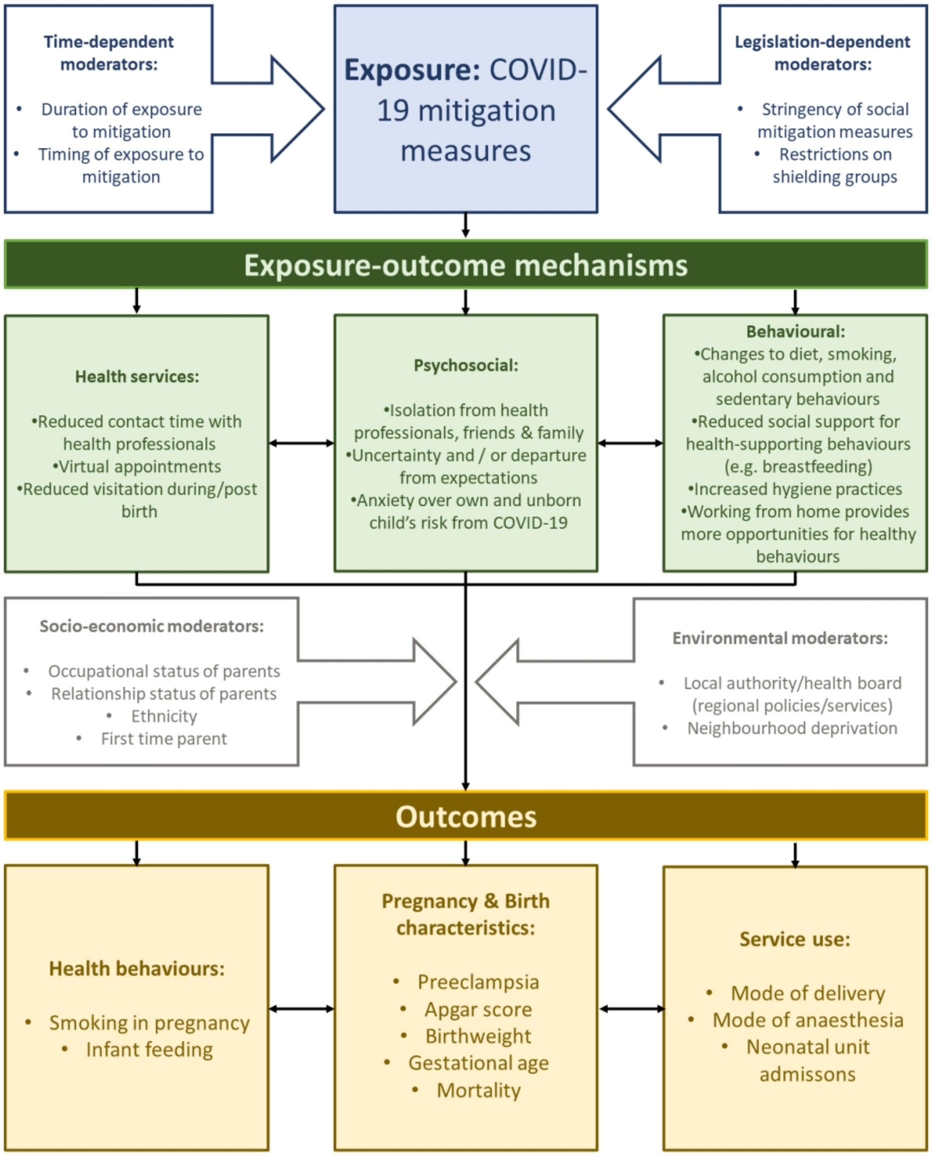

Figure 1: Logic model demonstrating the mechanisms and moderators of the wider impacts of the pandemic on pregnancy and birth outcomes

374x451mm (96 x 96 DPI)

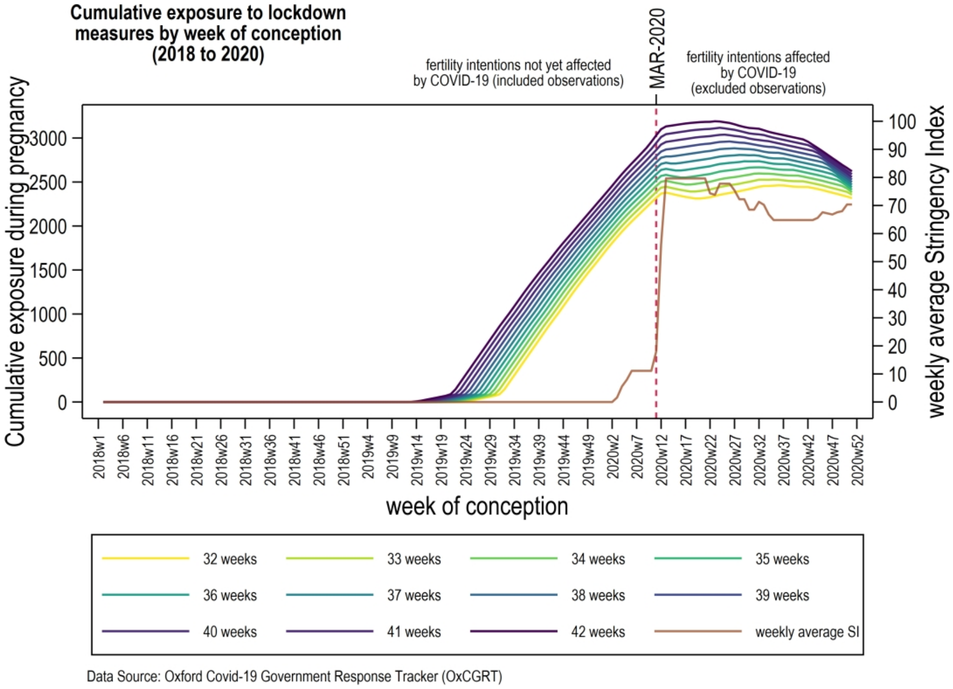

Figure 2: Visual description of our exposure variable. Sum of weekly average Stringency Index (left vertical axis) during pregnancy for each week of conception (42 weeks of gestation being the top line and 32 weeks of gestation being the bottom line) between January 2018 and December 2020. Level of cumulative exposure is shown for gestational age (32 to 42 weeks). Crude weekly average Stringency Index for Scotland is shown in brown (right vertical axis). Conceptions after March 2020 (indicated by the dashed red line) are excluded from our analyses.

1411x1058mm (72 x 72 DPI)

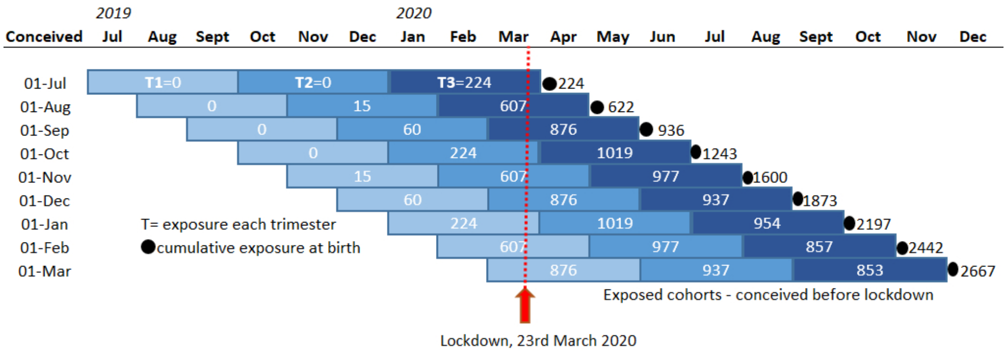

Figure 3: Exposed groups under investigation. Cumulative level of exposures presented here are the sum of weekly averages of the Stringency Index within each trimester up to month of birth. In this figure, conceptions and births are assumed to occur on the first of each month with equal gestational age. Note that, in the analyses, cumulative exposure is calculated for each mother-child pair individually and, thus, these exposure levels do not match those in Figure 2.

550x206mm (47 x 47 DPI)

The RECORD statement – checklist of items, extended from the STROBE statement, that should be reported in observational studies using routinely collected health data.

	Item No.	STROBE items	Location in manuscript where items are reported	RECORD items	Location in manuscript where items are reported
**Title and abstract**					
	1	(a) Indicate the study's design with a commonly used term in the title or the abstract (b) Provide in the abstract an informative and balanced summary of what was done and what was found	Page 1	RECORD 1.1: The type of data used should be specified in the title or abstract. When possible, the name of the databases used should be included.<br><br>RECORD 1.2: If applicable, the geographic region and timeframe within which the study took place should be reported in the title or abstract.<br><br>RECORD 1.3: If linkage between databases was conducted for the study, this should be clearly stated in the title or abstract.	Page 1 title and abstract<br><br>Page 1 abstract<br><br>Page 1 abstract
**Introduction**					
Background rationale	2	Explain the scientific background and rationale for the investigation being reported	Page 3		
Objectives	3	State specific objectives, including any prespecified hypotheses	Page 4,5<br>Figure 1		
**Methods**					
Study Design	4	Present key elements of study design early in the paper	Page 6		
Setting	5	Describe the setting, locations, and relevant dates, including periods of recruitment, exposure, follow-up, and data collection	Page 6,7,8,9		

For peer review only

Participants	6	*(a) Cohort study* - Give the eligibility criteria, and the sources and methods of selection of participants. Describe methods of follow-up<br>*Case-control study* - Give the eligibility criteria, and the sources and methods of case ascertainment and control selection. Give the rationale for the choice of cases and controls<br>*Cross-sectional study* - Give the eligibility criteria, and the sources and methods of selection of participants<br><br>*(b) Cohort study* - For matched studies, give matching criteria and number of exposed and unexposed<br>*Case-control study* - For matched studies, give matching criteria and the number of controls per case		RECORD 6.1: The methods of study population selection (such as codes or algorithms used to identify subjects) should be listed in detail. If this is not possible, an explanation should be provided.<br><br>RECORD 6.2: Any validation studies of the codes or algorithms used to select the population should be referenced. If validation was conducted for this study and not published elsewhere, detailed methods and results should be provided.<br><br>RECORD 6.3: If the study involved linkage of databases, consider use of a flow diagram or other graphical display to demonstrate the data linkage process, including the number of individuals with linked data at each stage.	Page 5, 6<br><br>Page 6,7
Variables	7	Clearly define all outcomes, exposures, predictors, potential confounders, and effect modifiers. Give diagnostic criteria, if applicable.	Page 7,8,9<br>Figure 2 and 3	RECORD 7.1: A complete list of codes and algorithms used to classify exposures, outcomes, confounders, and effect modifiers should be provided. If these cannot be reported, an explanation should be provided.	
Data sources/ measurement	8	For each variable of interest, give sources of data and details of methods of assessment (measurement).<br>Describe comparability of assessment methods if there is more than one group	Page 7,8,9		

For peer review only

Bias	9	Describe any efforts to address potential sources of bias	Page 8 to14		
Study size	10	Explain how the study size was arrived at	Page 15		
Quantitative variables	11	Explain how quantitative variables were handled in the analyses. If applicable, describe which groupings were chosen, and why	Page 7 to 13<br>Figure 2<br>Figure 3		
Statistical methods	12	(a) Describe all statistical methods, including those used to control for confounding<br>(b) Describe any methods used to examine subgroups and interactions<br>(c) Explain how missing data were addressed<br>(d) *Cohort study* - If applicable, explain how loss to follow-up was addressed<br>*Case-control study* - If applicable, explain how matching of cases and controls was addressed<br>*Cross-sectional study* - If applicable, describe analytical methods taking account of sampling strategy<br>(e) Describe any sensitivity analyses	Page 10 to 14		
Data access and cleaning methods		..		RECORD 12.1: Authors should describe the extent to which the investigators had access to the database population used to create the study population.	Page 6,7

For peer review only

				RECORD 12.2: Authors should provide information on the data cleaning methods used in the study.	
Linkage		..		RECORD 12.3: State whether the study included person-level, institutional-level, or other data linkage across two or more databases. The methods of linkage and methods of linkage quality evaluation should be provided.	Page 6,7
**Results**					
Participants	13	(a) Report the numbers of individuals at each stage of the study (*e.g.*, numbers potentially eligible, examined for eligibility, confirmed eligible, included in the study, completing follow-up, and analysed)<br>(b) Give reasons for non-participation at each stage.<br>(c) Consider use of a flow diagram	Not applicable – study protocol	RECORD 13.1: Describe in detail the selection of the persons included in the study (*i.e.,* study population selection) including filtering based on data quality, data availability and linkage. The selection of included persons can be described in the text and/or by means of the study flow diagram.	Not applicable – study protocol
Descriptive data	14	(a) Give characteristics of study participants (*e.g.*, demographic, clinical, social) and information on exposures and potential confounders<br>(b) Indicate the number of participants with missing data for each variable of interest<br>(c) *Cohort study* - summarise follow-up time (*e.g.*, average and total amount)	Not applicable – study protocol		
Outcome data	15	*Cohort study* - Report numbers of outcome events or summary measures over time<br>*Case-control study* - Report numbers in each exposure	Not applicable – study protocol		

For peer review only

		category, or summary measures of exposure<br>*Cross-sectional study* - Report numbers of outcome events or summary measures			
Main results	16	(a) Give unadjusted estimates and, if applicable, confounder-adjusted estimates and their precision (e.g., 95% confidence interval). Make clear which confounders were adjusted for and why they were included<br>(b) Report category boundaries when continuous variables were categorized<br>(c) If relevant, consider translating estimates of relative risk into absolute risk for a meaningful time period	Not applicable – study protocol		
Other analyses	17	Report other analyses done—e.g., analyses of subgroups and interactions, and sensitivity analyses	Not applicable – study protocol		
**Discussion**					
Key results	18	Summarise key results with reference to study objectives	Not applicable – study protocol		
Limitations	19	Discuss limitations of the study, taking into account sources of potential bias or imprecision. Discuss both direction and magnitude of any potential bias	Not applicable – study protocol	RECORD 19.1: Discuss the implications of using data that were not created or collected to answer the specific research question(s). Include discussion of misclassification bias, unmeasured confounding, missing data, and changing eligibility over time, as they pertain to the study being reported.	Not applicable – study protocol
Interpretation	20	Give a cautious overall interpretation of results considering objectives,	Not applicable – study protocol		

For peer review only

		limitations, multiplicity of analyses, results from similar studies, and other relevant evidence			
Generalisability	21	Discuss the generalisability (external validity) of the study results	Not applicable – study protocol		
**Other Information**					
Funding	22	Give the source of funding and the role of the funders for the present study and, if applicable, for the original study on which the present article is based	Page 15		
Accessibility of protocol, raw data, and programming code		Not applicable – study protocol		RECORD 22.1: Authors should provide information on how to access any supplemental information such as the study protocol, raw data, or programming code.	Not applicable – study protocol

*Reference: Benchimol EI, Smeeth L, Guttmann A, Harron K, Moher D, Petersen I, Sørensen HT, von Elm E, Langan SM, the RECORD Working Committee. The REporting of studies Conducted using Observational Routinely-collected health Data (RECORD) Statement. *PLoS Medicine* 2015; in press.

*Checklist is protected under Creative Commons Attribution (CC BY) license.

For peer review only
